# Myeloid miR-155 deficiency exacerbates viral encephalitis by hindering M1 macrophage polarization due to impaired NLRP3 inflammasome activation in extraneural tissues

**DOI:** 10.3389/fimmu.2026.1818106

**Published:** 2026-06-11

**Authors:** Hee Won Byeon, Jin Young Choi, Hye Won Cho, Hyo Jin Kim, Seong Ok Park, Erdenebelig Uyangaa, Koanhoi Kim, Seong Kug Eo

**Affiliations:** 1 Bio-Safety Research Institute, and Core Facility Center for Zoonosis Research (Core-FCZR), College of Veterinary Medicine, Jeonbuk National University, Iksan, Republic of Korea; 2 Department of Pharmacology, School of Medicine, Pusan National University, Yangsan, Republic of Korea

**Keywords:** CNS inflammation, M1 macrophage, miR-155, neurotrophic virus, NLRP3 inflammasome

## Abstract

**Introduction:**

miR-155 regulates diverse inflammatory responses; however, its role in neurotropic virus–induced CNS inflammation, particularly in specific immune cell subsets, remains unclear. This study aimed to identify the immune cell population in which miR-155 is required for protection against Japanese encephalitis virus (JEV) and to elucidate the underlying mechanisms.

**Methods:**

Conditional miR-155 knockout mice with cell type–specific deletion in myeloid cells, dendritic cells (DCs), T cells, or B cells were generated using Cre–LoxP technology. Following JEV infection, we assessed survival, viral burden, type I interferon responses, M1 macrophage polarization, and NLRP3 inflammasome activation. Downstream targets of miR-155 and the effects of NLRP3 inhibition or IL-1α/IL-1β neutralization were also evaluated.

**Results:**

Myeloid cell–specific deletion of miR-155, but not its deletion in other immune cell types, significantly increased susceptibility to JEV infection and exacerbated neuroinflammation. This phenotype was associated with elevated viral burdens in both lymphoid and CNS tissues, impaired type I interferon responses, defective M1 macrophage polarization, and reduced NLRP3 inflammasome activation. Mechanistically, Peli1, Jarid2, and Bcl6 were identified as key downstream targets of miR-155 that regulate M1 polarization and inflammasome activity. Furthermore, pharmacological inhibition of NLRP3 or neutralization of IL-1α and IL-1β further worsened disease severity and suppressed M1 polarization.

**Discussion:**

These findings demonstrate that miR-155 in myeloid cells, particularly macrophages, is essential for orchestrating protective immune responses during neurotropic virus–induced CNS inflammation by promoting M1 macrophage polarization and NLRP3 inflammasome activation.

## 1 Introduction

Viral encephalitis is a severe and often fatal inflammatory disease of the brain caused by neurotropic viruses, including herpes simplex virus, West Nile virus, and Japanese encephalitis virus (JEV), and is frequently associated with viral meningitis (1). Infection is typically initiated in peripheral tissues and subsequently spreads to the central nervous system (CNS) through hematogenous routes or retrograde axonal transport. Among these pathogens, Japanese encephalitis (JE) represents the most prevalent form of epidemic viral encephalitis worldwide, with a particularly high incidence in South and Southeast Asia, where an estimated 50,000–175,000 cases occur annually (2–5). As the leading arboviral cause of neurological disease, JE imposes a substantial global health burden, further exacerbated by the expanding geographic distribution of competent mosquito vectors and emerging evidence of mosquito-independent transmission (6–8). Following peripheral inoculation, JEV initially replicates in innate immune cells, including monocytes, macrophages, and dendritic cells (DCs), before disseminating systemically and crossing the blood–brain barrier to invade the CNS (9–13). Notably, disease severity is driven less by direct viral cytopathic effects and more by excessive and dysregulated host immune responses within the CNS. While virus-specific adaptive immunity, including neutralizing antibodies and T cell responses, contributes to viral clearance, its delayed activation necessitates a critical reliance on early innate immune responses. In particular, type I interferon (IFN-I) signaling plays a central role in restricting viral replication and spread during the initial phase of infection (13–16).

Macrophages are key regulators of this early immune response and serve as major sites of JEV replication, thereby facilitating viral amplification and dissemination to the CNS (17, 18). These cells exhibit remarkable functional plasticity, dynamically adopting distinct activation states in response to environmental cues. Classically activated (M1) macrophages promote antimicrobial and proinflammatory responses, whereas alternatively activated (M2) macrophages are associated with tissue repair and resolution of inflammation (19–22). During JEV infection, macrophages initiate innate immune responses through the production of inflammatory cytokines and chemokines, as well as antigen presentation in peripheral lymphoid tissues and within the CNS. While these responses are essential for controlling viral spread, they can also contribute to immunopathology when dysregulated (23–25). In addition, JEV-infected Ly-6C^+^ monocytes are recruited to the bloodstream and subsequently infiltrate the CNS, where they differentiate into inflammatory macrophages, DCs, and microglia-like cells, thereby amplifying neuroinflammation (26, 27). The balance between M1 and M2 macrophage polarization is therefore a critical determinant of disease outcome, influencing both antiviral defense and the extent of CNS damage. Moreover, JEV can actively modulate macrophage function by regulating suppressors of cytokine signaling (SOCS) proteins, which in turn affect JAK–STAT signaling pathways and alter cytokine production (25). These findings underscore the complex and context-dependent role of macrophages in JEV pathogenesis.

MicroRNAs (miRNAs) are small, single-stranded noncoding RNAs that regulate gene expression at the post-transcriptional level and play crucial roles in fine-tuning immune responses (28–30). Among them, miR-155, encoded by the B cell integration cluster (BIC), has emerged as a key regulator of innate and adaptive immunity during infection and inflammation (31). miR-155 expression is rapidly induced in macrophages and microglia in response to inflammatory stimuli such as lipopolysaccharide (LPS) and viral infection, where it modulates signaling pathways involved in cytokine production and antiviral defense (32, 33). It has also been implicated in the regulation of IFN signaling and NF-κB activation, both of which are critical for host responses to viral infections. In the context of JE, elevated levels of miR-155 have been detected in patient sera, and *in vitro* studies using microglial cells have demonstrated that miR-155 can influence both viral replication and inflammatory responses during JEV infection (34–36). For instance, miR-155 has been shown to target SHIP1, thereby promoting NF-κB–mediated proinflammatory cytokine production. Conversely, other studies suggest that miR-155 may exert anti-inflammatory or antiviral effects under certain conditions, highlighting its context-dependent functions. Despite these insights, most existing studies have relied on *in vitro* systems, and the *in vivo*, cell type–specific role of miR-155—particularly in macrophages—during JEV infection remains poorly understood.

To address this gap, we generated conditional knockout (cKO) mouse models in which miR-155 was selectively deleted in myeloid cells, CD11c^+^ DCs, CD4^+^/CD8^+^ T cells, and CD19^+^ B cells using the Cre–LoxP system (37). Notably, miR-155 deficiency in myeloid cells, but not in other immune cell populations, resulted in significantly increased susceptibility to JE and exacerbated neuroinflammation in the CNS. This phenotype was associated with elevated viral burden in both peripheral lymphoid tissues and the CNS, as well as impaired type I interferon responses. Mechanistically, the absence of miR-155 in myeloid cells disrupted M1 macrophage polarization and attenuated activation of the NLRP3 inflammasome. Further analyses identified *Peli1*, *Jarid2*, and *Bcl6* as key miR-155 target genes involved in regulating these processes. In addition, pharmacological inhibition of the NLRP3 inflammasome or neutralization of its effector cytokines, IL-1α and IL-1β, further increased susceptibility to JE and suppressed M1 macrophage polarization. Collectively, these findings demonstrate that myeloid miR-155 plays a critical role in promoting M1 macrophage polarization and NLRP3 inflammasome activation, thereby contributing to effective antiviral immunity and limiting JE progression.

## 2 Materials and methods

### 2.1 Ethics statement

All animal experiments described in the present study were conducted at Jeonbuk National University according to the guidelines set by the Institutional Animal Care and Use Committee (IACUC) of Jeonbuk National University and were pre-approved by the Ethics Committee for Animal Experiments of Jeonbuk National University (approval number: CBNU-2019-00079). The animal research protocol used in this study followed the guidelines set up by the nationally recognized Korea Association for Laboratory Animal Sciences (KALAS). All experimental protocols requiring biosafety were approved by the Institutional Biosafety Committee (IBC) of Jeonbuk National University and were performed in a biosafety cabinet at the Core Facility Center for Zoonosis Research, Jeonbuk National University.

### 2.2 Animals, cells, and viruses

Conditional knockout (cKO) mice lacking miR-155 expression in specific immune cell populations—miR155^ΔLysM^, miR155^ΔCD11c^, miR155^ΔCD4^, and miR155^ΔCD19^—were generated by cross-breeding miR155^fl/fl^ mice (C57BL/6-Mir155^tm1.1^Ggard/J, stock no. 026700) with LysM-Cre, CD11c-Cre, CD4-Cre, and CD19-Cre transgenic mice (The Jackson Laboratory, Bar Harbor, MA), respectively. In these models, miR-155 expression is specifically ablated in myeloid cells, CD11c^+^ DCs, CD4^+^/CD8^+^ T cells, and CD19^+^ B cells. OT-II transgenic mice, which express a T cell receptor (TCR) specific for the I-A^b^-restricted OVA_323–339_ peptide (ISQAVHAAHAEINEAGR) derived from chicken ovalbumin (OVA), were also obtained from The Jackson Laboratory. All mice were housed, screened, and bred in the animal facility at Jeonbuk National University. The JEV Beijing-1 strain was obtained from the Green Cross Research Institute (Suwon, Korea) and propagated in the mosquito cell line C6/36 (CRL-1660; ATCC, Manassas, VA) and BHK-21 cells (CCL-10; ATCC) using high-glucose Dulbecco’s Modified Eagle Medium (DMEM) supplemented with 2% fetal bovine serum (FBS), penicillin (100 U/ml), and streptomycin (100 U/ml), as described previously (9, 11). Viral stocks were titrated using a focus-forming assay and stored in aliquots at −80°C until use.

### 2.3 Antibodies and reagents

The fluorophore-conjugated antibodies used for the flow cytometric analysis and chemical reagents can be found in **Supplementary Table 1**. JEV epitope peptide of CD4^+^ T cells [NS1_132-145_, TFVVDGPETKECPD] and [NS3_563-574_, WCFDGPRTNAIL] or CD8^+^ T cells [NS4B_215-223_, SAVWNSTTA] was chemically synthesized at Peptron (Daejeon, Korea).

### 2.4 Mouse model of JE

miR155^fl/fl^ control mice and cKO mice—including miR155^ΔLysM^, miR155^ΔCD11c^, miR155^ΔCD4^, and miR155^ΔCD19^—were intraperitoneally (i.p.) infected with JEV at a dose of 2.5 × 10^7^ focus-forming units (FFU). Following infection, mice were monitored daily for survival, body weight loss, and neurological symptoms such as limb paralysis and reduced mobility with retained responsiveness. Clinical signs of encephalitis were evaluated daily using a previously established five-point scale (9, 11), defined as follows: (1) hunched posture and ruffled fur, (2) altered gait and reduced movement, (3) immobile but responsive, (4) moribund and unresponsive, and (5) death. Additionally, disease severity was categorized into seven clinical grades: subclinical, ruffled fur, hindlimb weakness, mild paralysis (rear leg paresis), moderate paralysis (paresis of both forelimbs and hindlimbs), severe paralysis, and moribund state (38).

### 2.5 Quantitative analysis of viral load and cytokine expression

#### 2.5.1 Quantitative qRT-PCR for viral load and cytokine expression

Viral load and cytokine/chemokine expression levels in inflammatory and lymphoid tissues, as well as in BMDM, were analyzed using SYBR Green- and probe-based qRT-PCR. Mice were infected with JEV (2.5 × 10^7^ FFU, i.p.), and tissues (spleen, brain, spinal cord) were harvested at designated time points. BMDM were infected with JEV at MOIs of 0.1, 1.0, and 5.0. Total RNA was extracted using Wizol™ reagent (Wizbiosolutions Inc., Korea), and reverse transcription was performed with a High-Capacity cDNA Reverse Transcription Kit (Wizbiosolutions Inc.). qRT-PCR was conducted using a CFX96 PCR System (Bio-Rad) with 2 μl of cDNA, 10 μl of 2× SYBR Premix Ex Taq, and 200 nM of gene-specific primers (**Supplementary Table 2**) in a 20 μl reaction volume. The cycling conditions were: 95°C for 30 s, followed by 45 cycles of 95°C for 5 s and 60°C for 20 s. Melting curve analysis (65–95°C, 0.2°C/15 s increments) confirmed product specificity. All reactions were performed in duplicate, with no-template controls included. Data were analyzed using Bio-Rad CFX Manager (v2.1); cytokine/chemokine levels were normalized to β-actin, and viral load was quantified by comparison to standard viral cDNA curves.

#### 2.5.2 Determinations of the production levels of cytokine protein

IL-1α and IL-1β levels were measured using sandwich ELISA kits (Invitrogen) with culture supernatants from JEV-infected BMDM and sera from infected mice. Nunc MaxiSorp 96-well plates (ThermoFisher, Waltham, MA) were coated with capture antibodies in coating buffer and incubated overnight at 4°C. After washing with PBST (PBS containing 0.05% Tween 20), plates were blocked with ELISA/ELISPOT diluents for 2 h at 37°C. Samples and cytokine standards were added and incubated overnight at 4°C. Biotin-conjugated detection antibodies were then applied for 1 h at 37°C, followed by streptavidin-HRP incubation. Color was developed using tetramethylbenzidine (TMB) substrate and stopped with 1M phosphoric acid. Absorbance was read at 450 nm (Molecular Devices), and cytokine concentrations were determined using SoftMax Pro 3.4 based on standard curves.

#### 2.5.3 Cytometric bead array

The levels of inflammatory cytokine proteins (IL-6, TNF-α, IL-10, CXCL1, CCL2, CXCL10) in serum samples were quantified using LEGENDplex™ (Biolegend, San Diego, CA), employing a Cytometric Bead Array (CBA) approach, in accordance with the manufacturer’s instructions. This technique facilitates the concurrent quantification of various cytokines within the serum, offering valuable information on the immune responses triggered under the experimental conditions being examined.

### 2.6 Histopathological examinations and confocal microscopy

Histopathological analyses were performed on brain tissues from miR155^fl/fl^ and miR155^ΔLysM^ cKO mice after JEV infection. The brain tissues were fixed in 10% neutral buffered formalin (NBF) (Clinic Medi Labs, Montreal, CA), embedded in paraffin, and then sectioned into 4-µm slices before being stained with hematoxylin and eosin (H&E). These sections were evaluated using a slide digital scanner (Motic, Kowloon, Hong Kong). For confocal microscopy, brain sections were deparaffinized and subjected to antigen retrieval using boiled Citration buffer (ScyTek Laboratories, West Logan, UT), followed by a gradual cooling at room temperature for 2 h. After rinsing with phosphate-buffered saline (PBS) for 5 minutes, sections were blocked with SuperBlock blocking buffer (ScyTek Laboratories). Primary antibodies targeting JEV NS1 and E proteins (diluted 1:20, Abcam), along with FITC-conjugated anti-mouse CD11b, were incubated with the sections overnight in humidified chambers at 4 °C. Following washes with PBS, secondary PE-conjugated goat anti-mouse IgG (SouthernBiotech, Birmingham, AL, USA) were applied sequentially to identify the bound primary antibodies. Nuclei were counterstained with DAPI (4’,6-diamidino-2-phenylindole; Sigma-Aldrich). Fluorescence images were acquired using a confocal laser scanning microscope (Carl Zeiss, Zena, Germany).

### 2.7 Analysis of infiltrated leukocytes in the CNS

Mice infected with JEV were perfused with heparin-supplemented Hank’s balanced salt solution (HBSS) via cardiac puncture of the left ventricle at the designated dpi as described earlier (9, 11). Brains were then harvested, gently homogenized by pressing them through a 100-mesh tissue sieve, and digested using 0.25 mg/ml collagenase type IV (Worthington Biochem, Freehold, NJ) and 10 mg/ml DNase I (Amresco, Solon, OH) in RPMI medium. This mixture was incubated for 1 h at 37°C with agitation. Cells were separated by centrifugation at 800×*g* for 30 min (Axis-Shield, Oslo, Norway) using Opti-prep density gradient (18/10/5%), and the cells were collected from 18% to 10% interface and washed twice with PBS. After counting, cells were labeled with directly conjugated Abs against CD45, CD11b and Ly-6C, Ly-6G or Tmem119 for 30 min at 4°C. Data collection and analysis were performed with a FACS Calibur flow cytometer (Becton Dickinson Medical Systems, Sharon, MA) and FlowJo (Tree Star, San Carlos, CA) software.

### 2.8 JEV-specific Igs production and T cell responses

JEV-specific IgM and IgG levels in sera were quantified by conventional ELISA using E glycoprotein Ag (PROSPEC, Rehovot, Israel). Concurrently, JEV-specific CD4^+^ and CD8^+^ T-cell responses were determined by intracellular IFN-γ and TNF-α staining in response to stimulation with JEV epitope peptides, as described previously (9). Surviving mice infected with JEV (2.5 × 10^7^ FFU) were euthanized at 7 dpi using a CO_2_ euthanasia system delivering an optimal flow rate to displace 30–70% of the chamber volume per minute, and splenic leukocytes were subsequently isolated. The splenocytes were then cultured in 96-well culture plates (1×10^6^ cells/well) in the presence of synthetic peptide epitopes (NS1_132–145_, NS3_563-574_, and NS4B_215–225_) for 12 h and 6 h to observe CD4^+^ and CD8^+^ T cell responses, respectively. Monensin at concentration of 2 μM was added to antigen-stimulated cells 6 h before harvest. Cells were washed twice with FACS buffer containing monensin, surface-stained with FITC-anti-CD4 or CD8 antibodies for 30 min at 4°C, and then washed twice with PBS containing monensin. After fixation, cells were washed twice with 1× Permeabilization Buffer (eBioscience) and stained with PepCP-Cy5.5 anti-IFN-γ or APC-anti-TNF-α in the permeabilization buffer for 30 min at room temperature. Finally, cells were washed twice with PBS and fixed using the fixation buffer. In some experiments, IFN-γ^+^CD4^+^ Th1, IL-4^+^CD4^+^ Th2, IL-17^+^CD4^+^ Th17 cells were analyzed by intracellular cytokine staining following brief stimulation with PMA and ionomycin (Sigma Aldrich, Burlington, MA). CD4^+^Foxp3^+^ Treg cells were enumerated by intracellular Foxp3 staining, combined with surface CD4 molecules. Sample analysis was performed using a FACS Calibur flow cytometer (Becton Dickson Medical Systems) with FlowJo software (Tree Star).

### 2.9 Immunoblot for inflammasome activation

BMDM cells derived from miR155^fl/fl^ and miR155^ΔLysM^ cKO mice were infected with JEV (5 MOI) and harvested at 6, 12, 24, 36, and 48 h post-infection. Harvested BMDM were lysed with PRO-PREP™ (iNtRON, Inc., Daejeon, Korea) and subsequently resolved cell proteins by electrophoresis on 10% SDS-polyacrylamide gels, followed by transfer to PVDF membranes. The membranes were blocked with 5% skim-milk (in TBST) for 1 h and probed with primary antibodies; anti-NLRP3, anti-ProCasp1/Casp1 and anti-β-actin at 4°C overnight. After four washes with TBST, the membranes were incubated for 2 h with HRP-conjugated secondary antibodies at room temperature. Bands were detected using ECL substrate solution and Chemi-Doc (Fusion Fx7, Vilber, FR).

### 2.10 Analysis of M1 macrophage polarization

M1 macrophage polarization was ex vivo evaluated by determining IL-12p40 and iNOS production in response to brief stimulation with LPS using leukocytes prepared from peritoneal cavity and spleen. Mice infected with JEV were euthanized to harvest the leukocytes from peritoneal cavity and spleens. Cells of peritoneal cavity were harvested using a solution of Hank’s balanced salt solution (HBSS) supplemented with 2% FBS, followed by two PBS washes. Splenocytes were harvested with RPMI medium containing 10% FBS, homogenized through pressing them on 100-mesh tissue sieve, and centrifuged at 800×g for 6 min. All cells were counted and stained for surface with CD45, CD11b, F4/80 and intracellularly stained with IL-12p40 or iNOS after brief stimulation with LPS. For M1 polarization of BMDM, BM cells were extracted from femur and tibia of miR155^fl/fl^ and miR155^ΔLysM^ mice, filtered to remove bone fragments with cell strainer. After that, BM cells were washed, resuspended, and incubated in culture medium with GM-CSF (10 ng/mL). GM-CSF was added four days-post incubation once more. Seven days post-incubation, the adherent BMDM were collected and seeded in an appropriate culture plate with RPMI1640 medium. After overnight incubation, the medium was replaced with fresh medium containing 100 ng/mL of LPS (Sigma-Aldrich, St. Louis, MO) and 10 ng/mL of murine IFN-γ (PEPROTECH, Texas, US) to induce M1 macrophage polarization. Following a12 h-incubation, BMDM was harvested for the analysis of M1 polarization.

### 2.11 Ag-presentation of M1-polarized BMDM

The Ag presentation capacity of M1-polarized BMDM derived from miR155^fl/fl^ and miR155^ΔLysM^ mice was evaluated based on their ability to present the OVA_323–339_ peptide to OT-II CD4^+^ T cells (39). Initially, CD4^+^ T cells were isolated from OT-II-Tg mice using the MojoSort isolation kit (Biolegend, San Diego, CA, USA), in accordance with the manufacturer’s guidelines. Subsequently, purified CD4^+^ T cells were co-cultured with either M1-polarized or JEV-infected BMDM at a 2:1 ratio in the presence of OVA_323–339_ peptide (2 µg/ml). The co-culture was then incubated for 24 h at 37°C in a humidified atmosphere containing 5% CO_2_. Upon harvesting cells from the co-culture system, the activation status of OT-II CD4^+^ T cells stimulated with OVA_323–339_ peptide-loaded BMDM was evaluated by staining surface markers (CD25, CD69) and performing intracellular staining of IL-2 and IFN-γ, in conjunction with surface CD4 staining. Data acquisition and analysis were conducted utilizing a FACS Calibur flow cytometer (Becton Dickinson Medical Systems) and FlowJo software (Tree Star).

### 2.12 Statistical analysis

All data are presented as mean ± standard error of the mean (SEM). Statistical differences between two groups were assessed using unpaired two-tailed Student’s *t*-test for ex vivo assays and immune cell analyses. For comparisons involving more than two groups, one-way or two-way analysis of variance (ANOVA) with repeated measures was performed, followed by Bonferroni *post hoc* tests. Viral burden and *in vivo* cytokine gene expression were analyzed using the Mann–Whitney *U* test or unpaired two-tailed *t*-test, as appropriate. A *p*-value ≤ 0.05 was considered statistically significant. All analyses were performed using GraphPad Prism 9.0.0 (GraphPad Software, San Diego, CA, USA).

## 3 Results

### 3.1 Myeloid miR-155 deficiency results in enhanced susceptibility to JE

**Figure 1:**
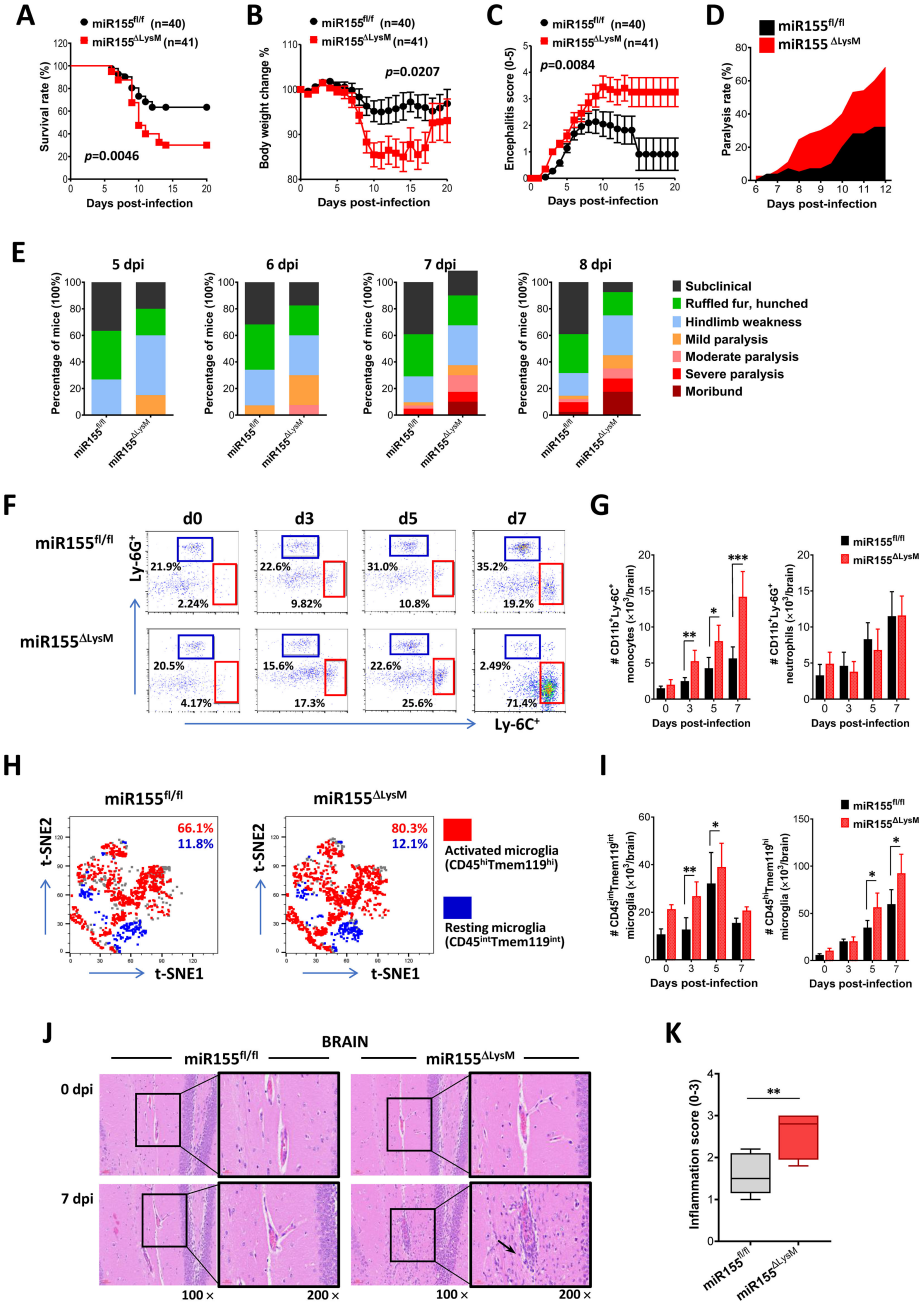
Myeloid miR-155 deficiency facilitates susceptibility to JE. **(A–D)** Susceptibility of miR155^fl/fl^ and miR155^ΔLysM^ cKO mice to JE progression. miR155^fl/fl^ control and miR155^ΔLysM^ cKO mice (n=40-41) were inoculated with JEV (2.5×10^7^ FFU) via i.p. route, and monitored over 20 days for their survival, body weight, encephalitis, and paralysis. **(A)** Curve showing survival rate. The proportion of surviving mice in each group was monitored daily for 20 days. **(B)** Changes in body weight. Data is expressed as the average ± SEM of body weight relative to the time of challenge. **(C)** Encephalitis score. Mice infected with JEV were expressed as the average score ± SEM of each group. **(D)** Ratio of mice showing neurological disorder during JE progression. Mice infected with JEV were examined every 12 h from 6 to 12 dpi, and the ratio of mice showing neurological disorder in infected mice was recorded. **(E)** Clinical signs. Clinical signs of JEV-infected miR155^fl/fl^ and miR155^ΔLysM^ cKO mice were monitored and categorized on day 5, 6, 7, and 8 post-infection. **(F)** CNS-infiltration kinetics of Ly-6C^+^ monocytes and Ly-6G^+^ neutrophils. **(G)** Total number of infiltrated Ly-6C^+^ monocytes and Ly-6G^+^ neutrophils in the brain. After vigorous heart perfusion, the frequency and total number of CD11b^+^Ly-6C^+^ monocytes and CD11b^+^Ly-6G^+^ neutrophils infiltrated into the brain were analyzed by flow cytometric analysis at the indicated days post-infection. The values in representative dot-plots show the average percentage of Ly-6C^+^ monocytes and Ly-6G^+^ neutrophils after gated on CD11b^+^ cells. **(H)** *t*-SNE maps describing the local probability of resting CD45^int^Tmem119^int^ and activated CD45^hi^Tmem119^hi^ microglia in the CNS. Representative *t*-SNE maps show the average percentages of resting CD45^int^Tmem119^int^ (blue) and activated CD45^hi^Tmem119^hi^ (red) microglia per group at 7 dpi. **(I)** Total number of resting CD45^int^Tmem119^int^ and activated CD45^hi^Tmem119^hi^ microglia in brain. The local probability and total number of resting and activated microglia were determined by flow cytometric analysis. **(J)** Representative photomicrographs of brain sections stained with H&E. Photomicrographs were taken from sagittal sections of the septo-striatal regions of brain derived from miR155^fl/fl^ and miR155^ΔLysM^ cKO mice at 7 dpi. The box denotes the area of interest. **(K)** Inflammation score of the brain tissues. Inflammation was scored based on the degree of infiltration of inflammatory cells at 7th dpi. Data in bar graphs denote the average ± SEM of the levels derived from at least three individual experiments (n=4-5). Statistical significance of body weight changes and encephalitis scores was determined using two-way repeated measures ANOVA, followed by Bonferroni *post hoc* tests. *p < 0.05; **p < 0.01; ***p < 0.001 between the levels derived from miR155^fl/fl^ control and miR155^ΔLysM^ cKO mice.

Despite the well-documented roles of miR-155 in various inflammatory responses, its impact on CNS inflammatory diseases induced by neurotrophic viral infections remains unreported. Notably, no studies have investigated the role of miR-155 expression in specific cell populations during the progression of fatal encephalitis caused by neurotropic viruses such as JEV. To explore the function of miR-155 in specific immune cell populations during JE progression, we generated miR155^ΔCD4^, miR155^ΔCD19^, miR155^ΔCD11c^, and miR155^ΔLysM^ cKO mice, in which miR-155 expression is selectively deficient in T cells, B cells, dendritic cells (DCs), and myeloid cells, respectively, through Cre-LoxP recombination (**Supplementary Figures 1A–D**) (37). These mice were then subjected to JEV infection to assess changes in susceptibility to JE. Our results revealed that miR-155 expression in DCs and myeloid cells plays a certain role in suppressing JE progression. Notably, miR155^ΔLysM^ cKO mice exhibited significantly increased susceptibility to JE following JEV infection (**Supplementary Figures 1E–M** and **Figure 1A**). While miR155^fl/fl^ control mice displayed a mortality rate of approximately 30%, miR155^ΔLysM^ cKO mice exhibited a significantly higher mortality rate of about 70%. In addition, miR155^ΔLysM^ cKO mice experienced a more pronounced weight loss compared to miR155^fl/fl^ control mice between days 9 and 18 following JEV infection (**Figure 1B**). The encephalitis scores were also observed to be higher in miR155^ΔLysM^ cKO mice, indicating more severe disease progression (**Figure 1C**). Notably, miR155^ΔLysM^ cKO mice developed neurological symptoms more rapidly and at a higher rate from day 6 post-infection compared to miR155^fl/fl^ control mice (**Figure 1D**). To clarify the clinical manifestations of JE in miR155^ΔLysM^ cKO mice, we monitored the clinical signs of JEV-infected mice, focusing on seven specific indicators in the course of JE. miR155^ΔLysM^ cKO mice developed obviously severe clinical signs from 5 to 8 dpi, compared to miR155^fl/fl^ control mice (**Figure 1E**). These results conclusively demonstrate that miR-155 deficiency in myeloid cells leads to increased susceptibility to JE, characterized by an escalation in the presentation of neurological disorders.

CNS-infiltration of Ly-6C^+^ monocytes and Ly-6G^+^ neutrophils derived from the myeloid cells represents a hallmark neuroinflammation in the progression of JE (18). To further characterize the CNS inflammation in miR155^ΔLysM^ cKO mice following JEV infection, we assessed the CNS infiltration of CD11b^+^Ly-6C^+^ monocytes and CD11b^+^Ly-6G^+^ neutrophils. Our results revealed a significantly increased frequency of infiltrated Ly-6C^+^ monocytes and Ly-6G^+^ neutrophils in the brain of miR155^ΔLysM^ cKO mice at 3, 5, and 7 dpi, in comparison to miR155^fl/fl^ control mice (**Figure 1F**). Notably, there was a 3- to 4-fold enhancement in the infiltration of Ly-6C^+^ monocytes at 7 dpi in miR155^ΔLysM^ cKO mice relative to miR155^fl/fl^ control mice. Similarly, a significant increase in the absolute number of infiltrated Ly-6C^+^ monocytes infiltrating the brain of miR155^ΔLysM^ cKO mice was noted at 3, 5, and 7 dpi (**Figure 1G**). Previous studies have established that microglia play a role in the pathogenesis of encephalitis caused by neurotrophic viruses, such as West Nile virus (40). Furthermore, it has been documented that CNS-infiltrating Ly-6C^+^ monocytes differentiate into inflammatory macrophages, including microglia (40). Consequently, we further examined the alterations of resting and activated microglia in the brain, based on the expression of Tmem119, a microglia-specific marker (41). As depicted in **Figure 1H**, at 7 dpi, miR155^ΔLysM^ cKO mice demonstrated a significantly higher frequency of CD11b^+^CD45^hi^Tmem119^hi^ activated microglia (80.3%) compared to miR155^fl/fl^ control mice (66.1%). In support, miR155^ΔLysM^ cKO mice contained accumulated number of CD11b^+^CD45^hi^Tmem119^hi^ activated microglia in the brain with higher levels at 5 and 7 dpi, relative to miR155^fl/fl^ control (**Figure 1I**). To better understand the severity of JE progression in miR155^ΔLysM^ cKO mice, we conducted a histopathological examination of brain tissues from miR155^fl/fl^ control and miR155^ΔLysM^ cKO mice post-JEV inoculation. As anticipated, miR155^ΔLysM^ cKO mice displayed pronounced CNS inflammation involving blood vessel, meninges, and ventricles in the brain compared to miR155^fl/fl^ control mice, based on CNS infiltration of peripheral leukocytes (**Figure 1J**). Supportively, miR155^ΔLysM^ cKO mice exhibited a higher CNS inflammation score compared to miR155^fl/fl^ control mice (**Figure 1K**). Collectively, these results suggest that the deficiency of miR155 expression in myeloid cells significantly exacerbates the progression of CNS inflammation, underscored by an augmented influx of inflammatory cells from peripheral sites following JEV infection.

### 3.2 Immune phenotypes of mice with myeloid cell-specific deficiency of miR-155 expression

Lysozyme M (LysM) is predominantly expressed in cells of the monocyte and granulocyte lineages (42). Consequently, in miR155^ΔLysM^ cKO mice, miR-155 expression is expected to be completely abrogated in neutrophils, reduced by approximately 83–90% in mature macrophages, and decreased by around 16% in DCs (43). To validate the reduction of miR-155 expression in specific myeloid subpopulations, various immune cell types were isolated from the spleen—including CD11b^+^F4/80^+^ mature macrophages, CD11c^+^ DCs, CD4^+^ and CD8^+^ T cells, and CD19^+^ B cells—and miR-155 expression following LPS stimulation was assessed. The results showed a substantial decrease in miR-155 expression in CD11b^+^F4/80^+^ mature macrophages in miR155^ΔLysM^ cKO mice (**Supplementary Figure 2A**). A modest reduction was also observed in CD11c^+^ DCs. In contrast, CD4^+^ and CD8^+^ T cells, as well as CD19^+^ B cells, exhibited miR-155 expression levels comparable to those in miR155^fl/fl^ control mice. These findings confirm that miR155^ΔLysM^ cKO mice exhibit a myeloid cell-specific reduction in miR-155 expression, particularly within the monocyte lineage. Immunophenotypic analysis of these mice revealed no significant differences in the numbers of splenic myeloid or lymphoid cells (**Supplementary Figure 2B**). Furthermore, serum immunoglobulin isotype levels remained unchanged (**Supplementary Figure 2C**), and histological examinations of the spleen, lung and colon also revealed no apparent abnormalities (**Supplementary Figure 2D**). Therefore, despite the targeted reduction of miR-155 expression in myeloid cells, miR155^ΔLysM^ cKO mice exhibit no overt immune or histopathological alteration under basal condition. These observations collectively suggest that the specific deficiency of miR-155 expression in the monocyte lineage of miR155^ΔLysM^ cKO mice increases their susceptibility to JEV infection, without affecting other aspects of immune phenotype.

### 3.3 Myeloid miR-155 deficiency increases viral load with impairment of early innate immunity

**Figure 2:**
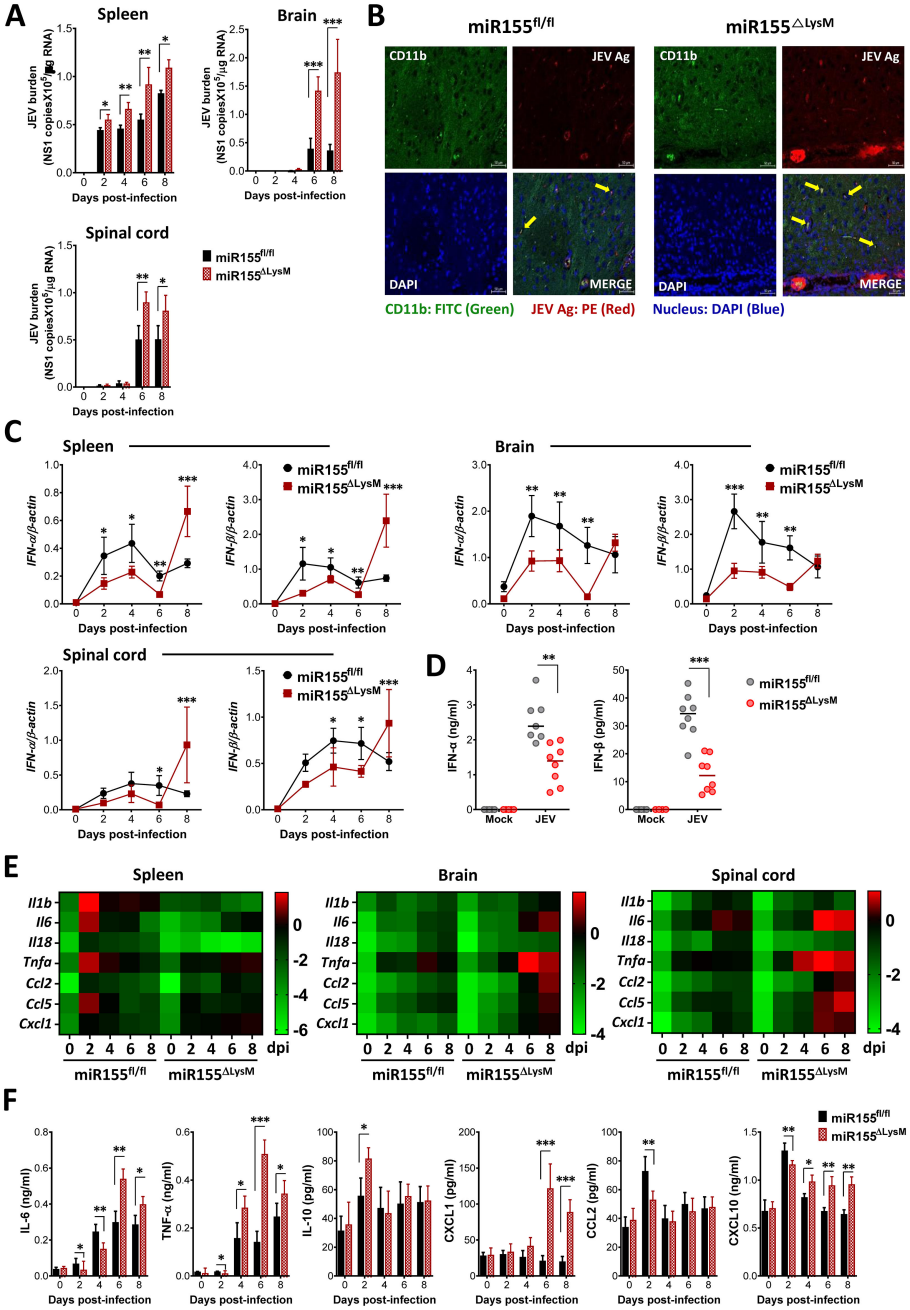
miR155^ΔLysM^ cKO mice show increased load of JEV with impairment of early innate immunity. **(A)** JEV burden in lymphoid and CNS tissues during the course of JE. Viral burden in the spleen, brain, and spinal cord of miR155^fl/fl^ control and miR155^ΔLysM^ cKO mice was assessed by qRT-PCR at the indicated days post-JEV infection. Viral RNA load was expressed as the number of viral RNA copies targeting the JEV NS1 gene per microgram of total RNA. **(B)** Representative confocal microscopic images. Brain sections prepared at 7 dpi were co-stained for JEV antigen (red), the nuclear stain DAPI (blue), and microglia/macrophage cell-marker CD11b (green). Yellow arrows indicate double-positive cells. **(C)** The expression of IFN-I (IFN-α/β) in lymphoid and CNS tissues of miR155^ΔLysM^ cKO mice during JE progression. The expression of IFN-α/β mRNA was determined by qRT-PCR at the indicated dpi. **(D)** Reduced production of IFN-I protein in sera of miR155^ΔLysM^ cKO mice at the early stage. The levels of IFN-α and IFN-β protein in sera were determined by cytokine ELISA at 1 dpi. **(E)** Enhanced expression of pro-inflammatory cytokines in the CNS tissues of miR-155^ΔLysM^ cKO mice at the late stage. The expression of pro-inflammatory cytokine mRNA was assessed by qRT-PCR using total RNA extracted from the spleen, brain, and spinal cord. Cytokine expression levels were normalized to the housekeeping gene β-actin and presented as the average of at least three independent samples on a log_2_ scale, with colors indicating relative expression levels. **(F)** Enhanced production of systemic inflammatory cytokines in sera of miR155^ΔLysM^ cKO mice. The levels of cytokine proteins in sera were determined by CBA at the indicated dpi. Data in bar and line graphs denote the average ± SEM of the levels derived from at least three individual experiments (n=4-5). *p < 0.05; **p < 0.01; ***p < 0.001 between the levels derived from miR155^fl/fl^ control and miR155^ΔLysM^ cKO mice.

To further investigate the increased susceptibility of miR155^ΔLysM^ cKO mice to JE, we analyzed the viral load in the spleen as well as CNS tissues, including the brain and spinal cord, following JEV infection. We observed that miR155^ΔLysM^ cKO mice exhibited progressively higher viral load in the spleen, brain, and spinal cord from day 2 to day 8 post-JEV infection, compared to miR155^fl/fl^ control mice (**Figure 2A**). Notably, the quantity of JEV presents in the CNS, specifically in the brain, was approximately five-fold higher in miR155^ΔLysM^ cKO mice than in miR155^fl/fl^ control mice on days 6 and 8 post-infection. This increase in JEV quantity within the brains of miR155^ΔLysM^ cKO mice was visualized using confocal microscopy. As anticipated, JEV Ag was detected more frequently in the brains of miR155^ΔLysM^ cKO mice compared to miR155^fl/fl^ control mice (**Figure 2B**). Furthermore, some of the JEV Ag colocalized with CD11b^+^ myeloid cells, highlighting a specific interaction within the CNS in response to JEV infection. IFN-I (IFN-α/β) innate responses are pivotal in curtailing viral proliferation during the initial stages of viral infection (11, 44). Consequently, to explore the association between elevated JEV load and IFN-I responses in miR155^ΔLysM^ cKO mice, we assessed the expression of IFN-I (IFN-α/β) in the spleen and CNS tissues (brain, spinal cord) following JEV infection. Up to 4 dpi, a critical period in the initial phase of infection, miR155^ΔLysM^ cKO mice exhibited lower expression of IFN-I (IFN-α/β) compared to miR155^fl/fl^ control mice (**Figure 2C**). However, in the later stages of infection, particularly after day 7, which coincides with the onset of neurological symptoms, an increase in IFN-I (IFN-α/β) expression was noted in the spleen, brain, and spinal cord of miR155^ΔLysM^ cKO mice. This increase is likely due to an intensified inflammatory response provoked by viral load in major inflamed tissues. Further examination of the amount of IFN-I (IFN-α/β) protein produced in the serum on day 1 post-infection revealed that miR155^ΔLysM^ cKO mice produced significantly lower levels of IFN-I (IFN-α/β) protein in comparison to miR155^fl/fl^ control mice (**Figure 2D**). Hence, it is inferred that miR155^ΔLysM^ cKO mice manifest a compromised initial IFN-I innate response, which may impede effective early viral clearance. Viral encephalitis is caused by excessive immune responses to neurotropic viruses, leading to dysregulated cytokine release and CNS damage (10), whereas balanced peripheral immune responses help clear the virus and prevent CNS invasion (11, 45). Therefore, analyzing cytokine and chemokine expression in both peripheral lymphoid and CNS tissues can offer valuable insights into JE progression in miR155^ΔLysM^ cKO mice. To gain a deeper understanding of JE progression in miR155^ΔLysM^ cKO mice, we examined the expression of cytokines and chemokines in the spleen and CNS tissues (brain, spinal cord) following JEV infection. Our results revealed that miR155^ΔLysM^ cKO mice exhibited reduced expression of cytokines and chemokines in the primary lymphoid tissue, the spleen, on day 2 post-JEV infection, compared to miR155^fl/fl^ control mice (**Figure 2E**). However, during the later stages of infection, specifically on days 6 and 8, miR155^ΔLysM^ cKO mice demonstrated a significant increase in the expression of inflammatory cytokines and chemokines within key inflamed tissues (brain, spinal cord). Furthermore, upon analyzing serum cytokine and chemokine levels, miR155^ΔLysM^ cKO mice initially produced somewhat lower levels of these proteins compared to miR155^fl/fl^ control mice but exhibited higher serum levels during the later stages of infection. (**Figure 2F**). Therefore, these findings suggest that the initial diminished expression of cytokines and chemokines in miR155^ΔLysM^ cKO mice leads to inadequate viral clearance, resulting in an increased viral load invading the CNS in later stages, thereby inducing a more pronounced inflammatory response within the CNS. In addition, the elevated expression of CCL2, CCL5, and CXCL1 in the later stages is likely to facilitate increased infiltration of peripheral leukocytes into the CNS.

### 3.4 miR-155 in myeloid cells needs to induce optimal acquired immune responses to JEV infection

**Figure 3:**
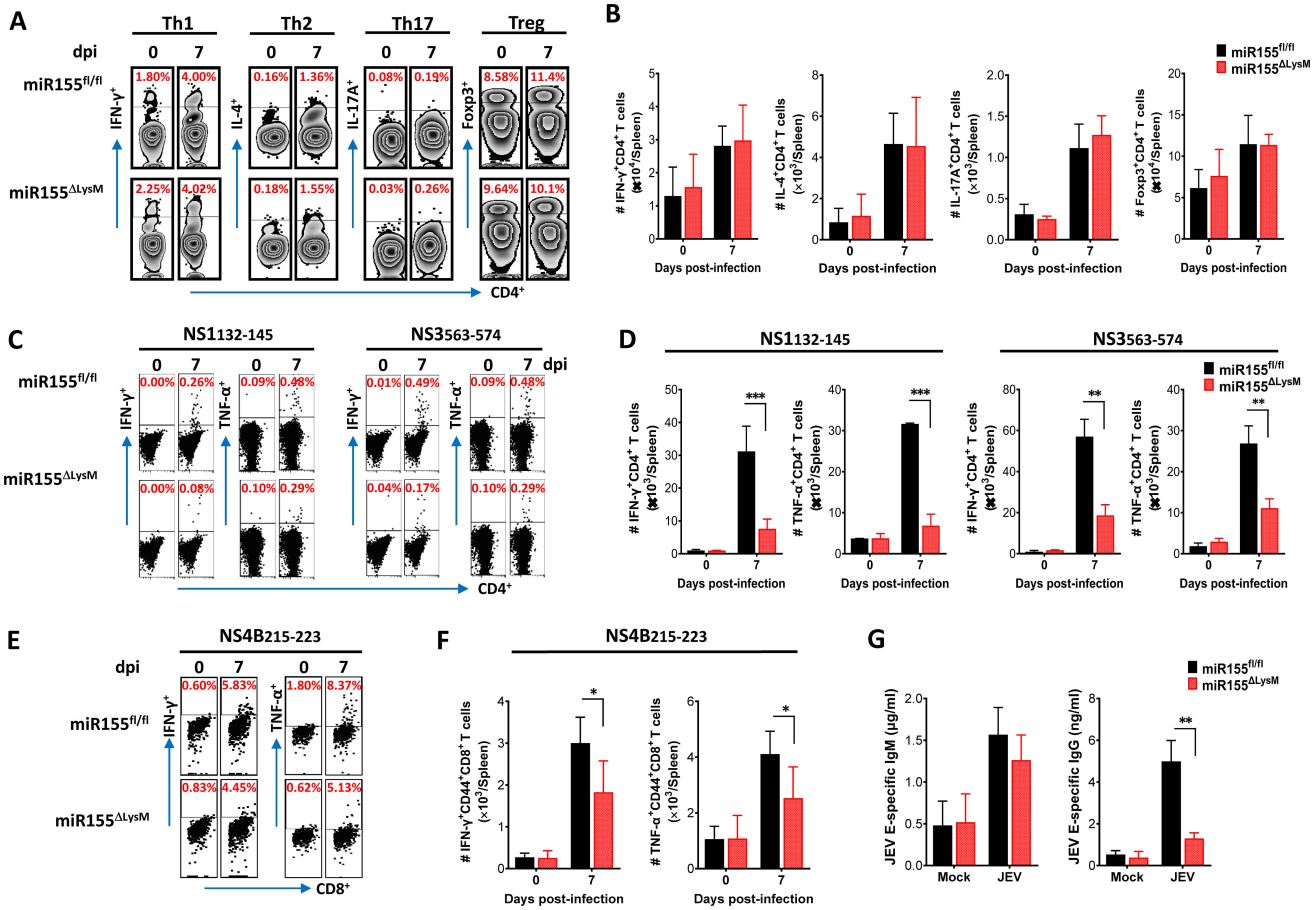
miR155^ΔLysM^ cKO mice exhibit impaired humoral and T-cell responses to JEV infection. **(A, B)** The frequency and total number of CD4^+^ Th1, Th2, Th17, and Foxp3^+^ Tregs in the spleen of miR155^fl/fl^ control and miR155^ΔLysM^ cKO mice. The frequency **(A)** and total number **(B)** of IFN-γ^+^CD4^+^ Th1, IL-4^+^CD4^+^ Th2, IL-17A^+^CD4^+^ Th17 were determined by intracellular cytokine staining with surface CD4 staining in response to brief stimulation with PMA and ionomycin at 7 dpi. CD4^+^Foxp3^+^ Tregs were analyzed by intracellular Foxp3 staining. **(C, D)** JEV-specific CD4^+^ T-cell responses. **(E, F)** JEV-specific CD8^+^ T-cell responses. The splenocytes were prepared from surviving miR155^fl/fl^ control and miR155^ΔLysM^ cKO mice 7 days post-JEV infection and used for stimulation with JEV epitope peptide of CD4^+^ T cells (NS1_132-145_, NS3_563-574_) or CD8^+^ T cells (NS4B_215-223_) for 12 or 6 h, respectively. The frequency **(C, E)** and absolute number **(D, F)** of JEV-specific CD4^+^ and CD8^+^ T cells were determined by intracellular cytokine (IFN-γ and TNF-α) staining combined with surface CD4 and CD8 staining, respectively. **(G)** Serum levels of JEV E protein-specific IgM and IgG. Levels of JEV E protein-specific IgM and IgG in sera were determined by conventional ELISA using sera collected from surviving mice at 7 dpi. Values in representative dot-plots denote the average percentage of indicated cell population. Data in bar graphs denote the average ± SEM of the levels derived from at least three individual experiments (n=3-4). *p < 0.05; **p < 0.01; ***p < 0.001 between the levels derived from miR155^fl/fl^ control and miR155^ΔLysM^ cKO mice.

Effector Ag-specific CD4^+^ and CD8^+^ T cells drive antiviral adaptive immune responses essential for controlling JE progression by eliminating JEV in extraneural tissues and the CNS (9). Despite some mice from both miR155^fl/fl^ control and miR155^ΔLysM^ cKO mice displaying neurological disorders at 5–6 days post-JEV infection – prior to full induction of functional adaptive immune responses, we examined the generation of JEV-specific CD4^+^ and CD8^+^ T-cell responses in the spleen of the survivors from both groups at 7 dpi. Upon analyzing the polyclonal CD4^+^ Th subsets with stimulation with PMA and ionomycin, it was found that no significant differences were noted in the distribution and overall numbers of IFN-γ^+^CD4^+^ Th1, IL-4^+^CD4^+^ Th2, and CD4^+^Foxp3^+^ Treg cells between miR155^ΔLysM^ cKO and miR155^fl/fl^ control mice, except that the IL-17^+^CD4^+^ Th17 subset exhibited a minor increase in miR155^ΔLysM^ cKO mice (**Figures 3A, B**). However, miR155^ΔLysM^ cKO mice displayed significantly diminished JEV-specific T cell responses in comparison to miR155^fl/fl^ control mice. Specifically, miR155^ΔLysM^ cKO mice possessed merely a quarter of IFN-γ-producing CD4^+^ T cells responsive to JEV NS1_132–145_ and NS3_563–574_ CD4^+^ T cell epitope peptides, and TNF-α-producing CD4^+^ T cells were halved relative to miR155^fl/fl^ control mice (**Figure 3C**). As a result, total count of IFN-γ and TNF-α-producing JEV-specific CD4^+^ T cells in the spleens of miR155^ΔLysM^ cKO mice was markedly low in response to stimulation with these peptides (**Figure 3D**). Moreover, upon evaluating JEV-specific CD8^+^ T cell response to NS4B_215–223_ CD8^+^ T cell epitope peptide, miR155^ΔLysM^ cKO mice also demonstrated a decreased distribution and total count of IFN-γ and TNF-α-producing JEV-specific CD8^+^ T cells in the spleen compared to miR155^fl/fl^ control mice (**Figures 3E, F**). Analysis of humoral immune responses to JEV infection revealed slightly lower IgM levels against JEV E protein in miR155^ΔLysM^ cKO mice, but the levels of JEV E-specific IgG were notably low (**Figure 3G**). This implies that the deficiency of miR155 in myeloid cells may influence JEV-specific IgG isotype switching after JEV infection. In conclusion, these findings demonstrate that the lack of miR-155 in myeloid cells leads to a weakened JEV-specific CD4^+^ and CD8^+^ T-cell response and could impede the induction of humoral immune responses following JEV infection.

### 3.5 miR-155 in myeloid cells is required for accumulation of M1 macrophages in inflamed tissues at the early stage during JE progression

**Figure 4:**
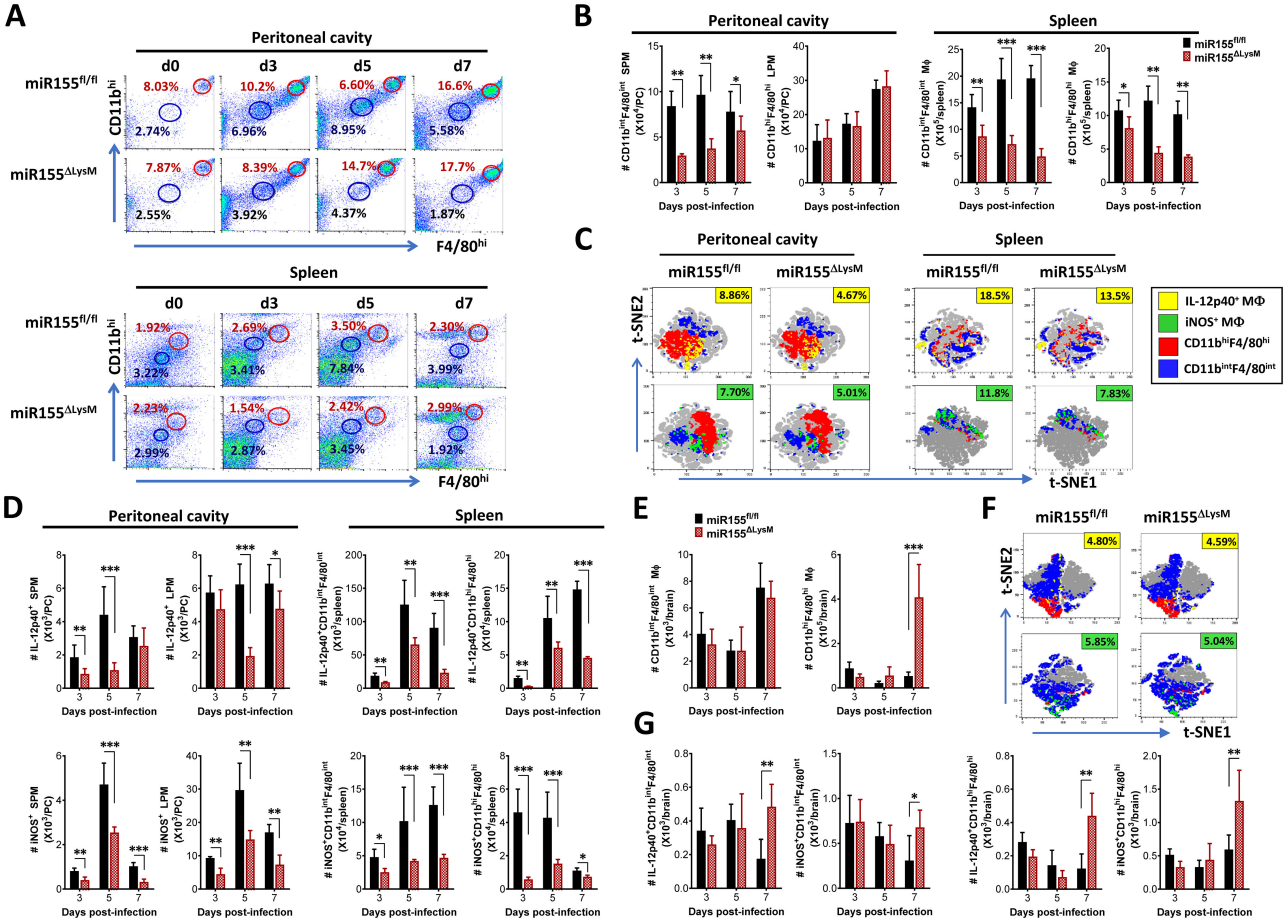
miR155^ΔLysM^ cKO mice exhibit reduced accumulation of M1-polarized macrophages in extraneural tissues following JEV infection. **(A)** Alteration of CD11b^+^F4/80^+^ macrophage subpopulations in peritoneal cavity (PC) and spleen during JE progression. **(B)** Total number of CD11b^int^F4/80^int^ and CD11b^hi^F4/80^hi^ macrophages in PC and spleen. The frequency and total number of CD11b^int^F4/80^int^ and CD11b^hi^F4/80^hi^ macrophages were analyzed by flow cytometric analysis. The values in representative dot-plots show the average percentages of CD11b^int^F4/80^int^ and CD11b^hi^F4/80^hi^ macrophages. **(C)** *t*-SNE maps describing the probability of IL-12p40 and iNOS-producing M1 macrophages in PC and spleen. Representative *t*-SNE maps show the average percentages of IL-12p40 and iNOS-producing M1 macrophages (CD11b^int^F4/80^int^ and CD11b^hi^F4/80^hi^ macrophages) per group at 5 dpi. **(D)** Total number of IL-12p40^+^ and iNOS^+^ M1 macrophages in PC and spleen. The total number of IL-12p40^+^ and iNOS^+^ M1 macrophages were determined by intracellular cytokine staining combined with surface markers CD11b and F4/80 in response to brief LPS stimulation using leukocytes prepared from PC and spleen. **(E)** Total number of CD11b^+^F4/80^+^ macrophage subpopulations in the brain during JE progression. **(F)** *t*-SNE maps describing the probability of IL-12p40 and iNOS-producing M1 macrophages in the brain. Representative *t*-SNE maps show the average percentages of IL-12p40 and iNOS-producing M1 macrophages (CD11b^int^F4/80^int^ and CD11b^hi^F4/80^hi^ macrophages) per group at 5 dpi. **(G)** Total number of IL-12p40^+^ and iNOS^+^ M1 macrophages in brain. After vigorous heart perfusion, total number of IL-12p40 and iNOS-producing M1 macrophages (CD11b^int^F4/80^int^ and CD11b^hi^F4/80^hi^ macrophages) in the brain were analyzed by flow cytometric analysis using intracellular cytokine staining. Data in bar graphs denote the average ± SEM of the levels derived from at least three individual experiments (n=3-4). *p < 0.05; **p < 0.01; ***p < 0.001 between the levels derived from miR155^fl/fl^ control and miR155^ΔLysM^ cKO mice.

Macrophages, diverse immune cells derived from Ly-6C^+^ and Ly-6C^-^ monocytes, are pivotal in neuroinflammation induced by neurotropic viruses (18, 40). Following viral infection, Ly-6C^+^ monocytes differentiate into either M1 or M2 macrophages based on the local microenvironment at sites of inflammation, influencing the course of viral-induced inflammation (21, 22). Accumulating evidence demonstrate that the expression of miR-155 in macrophages contributes to macrophage differentiation and function through diverse mechanisms (46). Therefore, to investigate the link between the deficiency of miR-155 expression in myeloid cells, including macrophages, and the increased susceptibility of miR155^ΔLysM^ cKO mice to JE, we examined the differentiation and function of macrophages in the peritoneal cavity and spleen during the early stages of JEV infection. The peritoneal cavity contains CD11b^hi^F4/80^hi^ large peritoneal macrophages (LPM) and CD11b^int^F4/80^int^ small peritoneal macrophages (SPM), the latter of which increase in response to inflammatory signals (47). Before JEV infection, CD11b^hi^F4/80^hi^ LPM predominates in the peritoneal cavity, but an increase in CD11b^int^F4/80^int^ SPM was observed following JEV infection via i.p route (**Figure 4A**). miR155^ΔLysM^ cKO mice displayed a markedly reduced increase in CD11b^int^F4/80^int^ SPM post-infection compared to miR155^fl/fl^ control mice. Similarly, although there was an increase in CD11b^int^F4/80^int^ macrophages in the spleen of miR155^fl/fl^ control mice following JEV infection, miR155^ΔLysM^ cKO mice showed a limited increase in these macrophages, even demonstrating a decrease by day 7 post-infection. When examining the total counts of CD11b^int^F4/80^int^ and CD11b^hi^F4/80^hi^ macrophages in the peritoneal cavity and spleen, excluding peritoneal CD11b^hi^F4/80^hi^ LPM, miR155^ΔLysM^ cKO mice had lower levels of macrophages compared to miR155^fl/fl^ control mice (**Figure 4B**). During inflammation, macrophages undergo differentiation into M1 or M2 phenotypes based on the microenvironment, thus exerting specific functions (21, 22). Consequently, we examined the M1 polarization of CD11b^int^F4/80^int^ and CD11b^hi^F4/80^hi^ macrophages in the peritoneal cavity and spleen following JEV infection. Our findings revealed a lower presence of M1-polarized macrophages in both the peritoneal cavity and spleen of miR155^ΔLysM^ cKO mice. Specifically, upon analyzing IL-12p40 and iNOS-producing M1-polarized macrophages after brief LPS stimulation, miR155^ΔLysM^ cKO mice were found to have lower levels of M1 macrophages than miR155^fl/fl^ control mice (**Figure 4C**). Similarly, total count of IL-12p40 and iNOS-producing M1 macrophages in both peritoneal cavity and spleen was also reduced in miR155^ΔLysM^ cKO mice (**Figure 4D**). However, analysis of the distribution of CD11b^int^F4/80^int^ and CD11b^hi^F4/80^hi^ macrophages in the brain revealed no significant difference in CD11b^int^F4/80^int^ macrophages between miR155^fl/fl^ control and miR155^ΔLysM^ cKO mice, but an increase in CD11b^hi^F4/80^hi^ macrophages was observed in miR155^ΔLysM^ cKO mice on day 7 post-infection (**Figure 4E**). Further examination of M1 macrophage polarization in the brain indicated a slight increase in M1 macrophages in miR155^ΔLysM^ cKO mice compared to miR155^fl/fl^ control mice on day 5 post-infection (**Figure 4F**). Similarly, total count of IL-12p40 and iNOS producing M1 macrophages in the brain also showed greater accumulation in miR155^ΔLysM^ cKO mice from day 5 post-infection, aligning with the onset of neurological symptoms (**Figure 4G**). These results indicate that the early suppression of M1 macrophage polarization in extraneural peripheral sites in miR155^ΔLysM^ cKO mice may lead to inadequate control of viral replication after JEV infection. Consequently, this insufficient viral containment exacerbates neuroinflammation in the CNS tissues during later infection stages, driven by an increased viral load. This sequence of events culminates in a heightened presence of M1 macrophages in the brains of miR155^ΔLysM^ cKO mice.

### 3.6 Macrophages derived from miR155^ΔLysM^ cKO mice show attenuated M1-polarization with reduced production of IL-1α and IL-1β during JE progression

**Figure 5:**
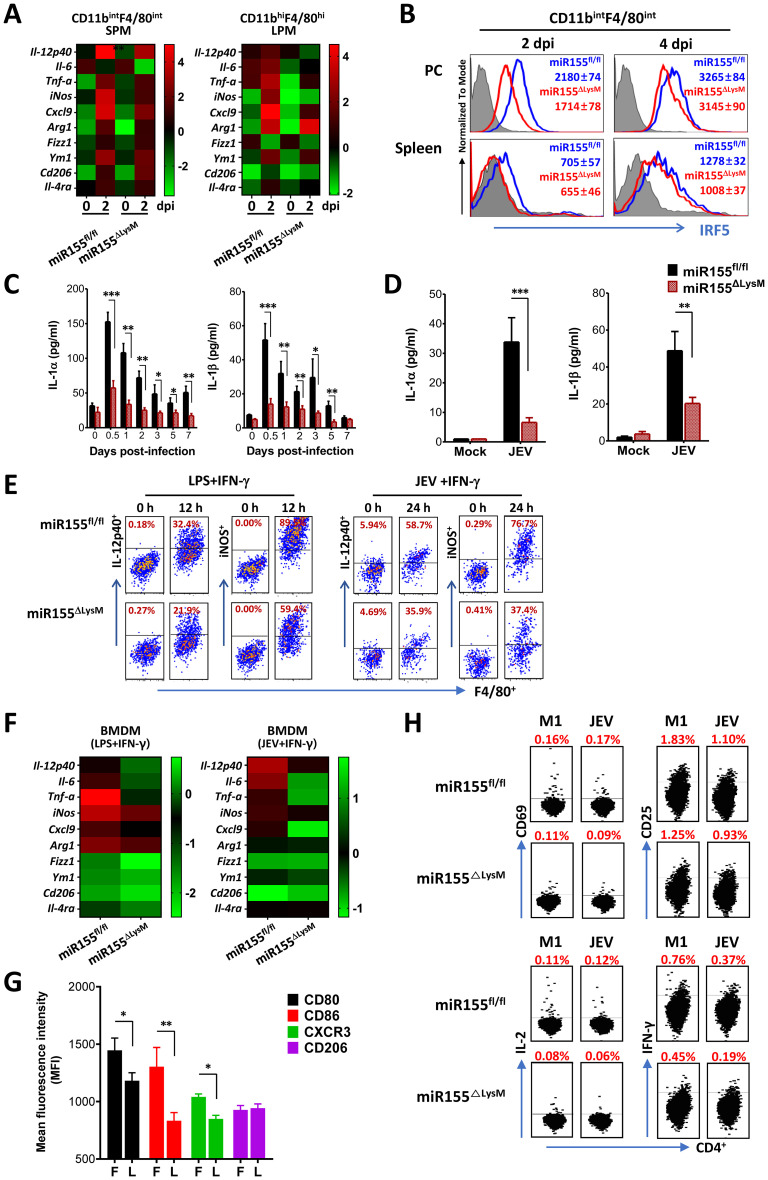
Macrophages derived from miR155^ΔLysM^ cKO mice show attenuated M1-polarization with reduced production of IL-1α and IL-1β during JE progression. **(A)** The expression of effector molecules for M1 and M2 macrophages derived from the peritoneal cavity. Two days following JEV infection, CD11b^int^F4/80^int^ SPM and CD11b^hi^F4/80^hi^ LPM sorted from the peritoneal cavity (PC) of miR155^fl/fl^ control and miR155^ΔLysM^ cKO mice were employed to determine the expression of effector molecule mRNA using qRT-PCR. The expression levels of effector molecules were normalized to the housekeeping gene β-actin and presented as the average of at least three independent samples on a log_2_ scale, with colors indicating relative expression levels. **(B)** IRF5 expression in CD11b^int^F4/80^int^ macrophages derived from the PC and spleen. The expression level of IRF5 in CD11b^int^F4/80^int^ macrophages derived from the PC and spleen of miR155^fl/fl^ control and miR155^ΔLysM^ cKO mice was determined by intracellular transcription factor staining combined with surface CD11b and F4/80 staining 2 and 4 dpi. The values in histograms denote the average ± SEM of IRF5 mean fluorescence intensity (MFI) in CD11b^int^F4/80^int^ SPM and CD11^int^F4/80^int^ splenic macrophages. **(C)** Reduced production of IL-1α and IL-1β in sera of miR155^ΔLysM^ cKO mice during JE progression. The levels of IL-1α and IL-1β proteins in sera were determined by cytokine ELSIA at the indicated dpi. **(D)** Reduced production of IL-1α and IL-1β in miR-155-deficient BMDM by JEV infection. Macrophages derived from BM cells of miR155^fl/fl^ control and miR155^ΔLysM^ cKO mice were infected with JEV (5 MOI), and the IL-1α and IL-1β protein levels were assessed by cytokine ELISA using culture media at 1 dpi. **(E)** Impaired M1 polarization of BMDM derived from miR155^ΔLysM^ cKO mice. BMDM prepared from miR155^fl/fl^ control and miR155^ΔLysM^ cKO mice was stimulated with LPS and IFN-γ or infected with JEV in the presence of IFN-γ for 12 h or 48 h, respectively. The M1 polarization of macrophages was assessed by intracellular cytokine staining for IL-12p40 and iNOS. **(F)** Impaired expression of M1 effector molecules in miR-155-deficient BMDM. BMDM prepared from miR155^fl/fl^ control and miR155^ΔLysM^ cKO mice were with LPS and IFN-γ or infected with JEV in the presence of IFN-γ, and used for determining the expression of M1 effector molecules with qRT-PCR. The expression levels of effector molecules were normalized to the housekeeping gene β-actin and presented as the average of at least three independent samples on a log_2_ scale, with colors indicating relative expression levels. **(G)** The expression of M1 and M2 surface markers in BMDM derived from miR155^fl/fl^ control and miR155^ΔLysM^ cKO mice. BMDM prepared from miR155^fl/fl^ control **(F)** and miR155^ΔLysM^ cKO mice **(L)** were stimulated with LPS and IFN-γ, and used for evaluating the expression of surface markers (CD80, CD86, CXCR3, CD206) by flow cytometric analysis. Values in the histograms show the average MFI ± SEM of each surface molecule expression. **(H)** Ag-presenting ability of miR155-deficient macrophages. BMDM generated from the BM cells of miR155^fl/fl^ and miR155^ΔLysM^ mice were either stimulated with LPS and IFN-γ (M1) or infected with JEV in the presence of IFN-γ (JEV). These BMDM were then co-cultured with purified OT-II CD4^+^ T cells in the presence of the OVA_3_;_23_;–_339_ peptide for 24 hours following a washing step. The Ag-presentation capacity of BMDMs to OT-II CD4^+^ T cells was evaluated by intracellular staining for IL-2 and IFN-γ, as well as surface staining for activation markers CD25 and CD69, combined with CD4 surface staining. The values in representative dot-plots show the average of positive cells for CD25, CD69, IL-2, and IFN-γ after gating on CD4^+^ cells. Data in bar graphs denote the average ± SEM of the levels derived from at least three individual experiments (n=3-4). *p < 0.05; **p < 0.01; ***p < 0.001 between the levels derived from miR155^fl/fl^ control and miR155^ΔLysM^ cKO mice.

To gain a deeper understanding of suppressed M1 macrophage polarization in miR155^ΔLysM^ cKO mice, we investigated the expression of effector molecules of M1/M2 macrophages in CD11b^int^F4/80^int^ SPM and CD11b^hi^F4/80^hi^ LPM isolated from the peritoneal cavity after JEV infection. SPM and LPM derived from miR155^ΔLysM^ cKO mice showed inhibited expression of M1 macrophage effector molecules, including IL-12p40, IL-6, TNF-α, iNOS, and CXCL9, with a notably more pronounced suppression in LPM (**Figure 5A**). However, the expression of M2 macrophage effector molecules did not differ in SPM and LPM between miR155^fl/fl^ control and miR155^ΔLysM^ cKO mice. IRF4 is known for a transcription factor involved in M2 macrophage polarization, while IRF5 is known to participate in M1 macrophage polarization (48). Analysis of IRF5 expression revealed a reduction in peritoneal CD11b^int^F4/80^int^ SPM and splenic CD11b^int^F4/80^int^ macrophages from miR155^ΔLysM^ cKO mice (**Figure 5B**). These observations suggest that miR-155 deficiency in macrophages may impair M1 macrophage differentiation during JE progression following JEV infection.

Recent research has illuminated the impact of inflammasome activation in modulating macrophage polarization into M1 and M2 phenotypes, thereby significantly affecting diseases progression (49). The subsequent investigation was undertaken to elucidate the link between inflammasome activation and diminished M1 macrophage polarization in miR155^ΔLysM^ cKO mice by analyzing the levels of IL-1α and IL-1β, the terminal effector molecules of inflammasome activation, in sera at the earl stage after JEV infection. Our results demonstrated that miR155^ΔLysM^ cKO mice produced considerably lower levels of IL-1α and IL-1β in their serum compared to miR155^fl/fl^ control mice (**Figure 5C**). Further analyses of BMDM derived from both miR155^fl/fl^ control and miR155^ΔLysM^ cKO mice revealed a marked reduction in IL-1α and IL-1β production in miR155-deficient BMDM after JEV infection (**Figure 5D**). In addition, BMDM derived from miR155^ΔLysM^ cKO mice exhibited significantly weakened M1 macrophage polarization in response to LPS or JEV and IFN-γ stimulation compared to those derived from miR155^fl/fl^ control mice (**Figure 5E**). Examination of M1/M2 macrophage effector molecule expression indicated a reduction in M1 macrophage effector molecules (IL-12p40, IL-6, TNF-α, iNOS, CXCL9) in BMDM derived from miR155^ΔLysM^ cKO mice under stimulation with LPS or JEV and IFN-γ, with no observed change in M2 macrophage effector molecule expression (**Figure 5F**). Furthermore, the surface expression of molecules highly expressed in M1 macrophages, such as CD80, CD86, and CXCR3, was found to be notably lower in BMDM derived from miR155^ΔLysM^ cKO mice (L) compared to those from miR155^fl/fl^ (F) control mice (**Figure 5G**). Given that molecules associated with Ag presentation (CD80, CD8) are upregulated in M1-polarized macrophages (48), further study assessed the Ag-presenting capacity of BMDM derived from miR155^fl/fl^ control and miR155^ΔLysM^ cKO mice through co-culture experiments with purified OT-II CD4^+^ T cells in the presence of OVA_323–339_ peptide. As anticipated, BMDM derived from miR155^ΔLysM^ cKO mice demonstrated a weaker activation of OT-II CD4^+^ T cells compared to those from miR155^fl/fl^ control mice (**Figure 5H**). Collectively, these findings suggest that miR155^ΔLysM^ cKO mice exhibit impaired inflammasome activation in macrophages following JEV infection, leading to weakened M1 macrophage polarization and a subsequent reduction in the antigen-presenting capacity to CD4^+^ T cells.

### 3.7 miR-155-deficient macrophages exhibit impaired activation of the NLRP3 inflammasome along with increased JEV replication

miRNAs, including miR-155, regulate the expression of mRNAs, while each mRNA can be targeted by various miRNAs (28). To identify potential miR-155 target genes involved in the suppression of M1 polarization and inflammasome activation in macrophages, we stimulated BMDM from miR155^fl/fl^ control and miR155^ΔLysM^ cKO mice with LPS and IFN-γ, followed by analysis of transcription factor expression. This analysis revealed that the absence of miR-155 led to differential regulation of several transcription factors (**Supplementary Figure 3A**). Specifically, miR-155-deficient BMDM exhibited upregulated expression of Peli1, STAT3, GATA3, STAT6, Bcl6, and Jarid2, whereas c-Maf and PU.1 expression was downregulated (**Supplementary Figure 3B**). Furthermore, BMDM infected with JEV and IFN- γ also displayed altered transcription factor profiles (**Supplementary Figure 4A**). Notably, Peli1, Bcl6, and Jarid2, which were elevated in response to LPS and IFN-γ stimulation, remained upregulated, whereas GATA3 expression decreased at 24 h post-JEV infection in miR-155-deficient BMDM (**Supplementary Figure 4B**). These findings indicate that the expression of candidate miR-155 target genes is modulated in a stimulus-dependent manner. Importantly, Peli1, Jarid2, and Bcl6 harbor putative miR-155 binding sites (**Supplementary Figure 4C**), suggesting they are direct regulatory targets of miR-155. The dysregulation of these genes in the absence of miR-155 likely contributes to altered M1 macrophage polarization and inflammasome activation, underscoring the multifaceted role of miR-155 in modulating the inflammatory response.

**Figure 6:**
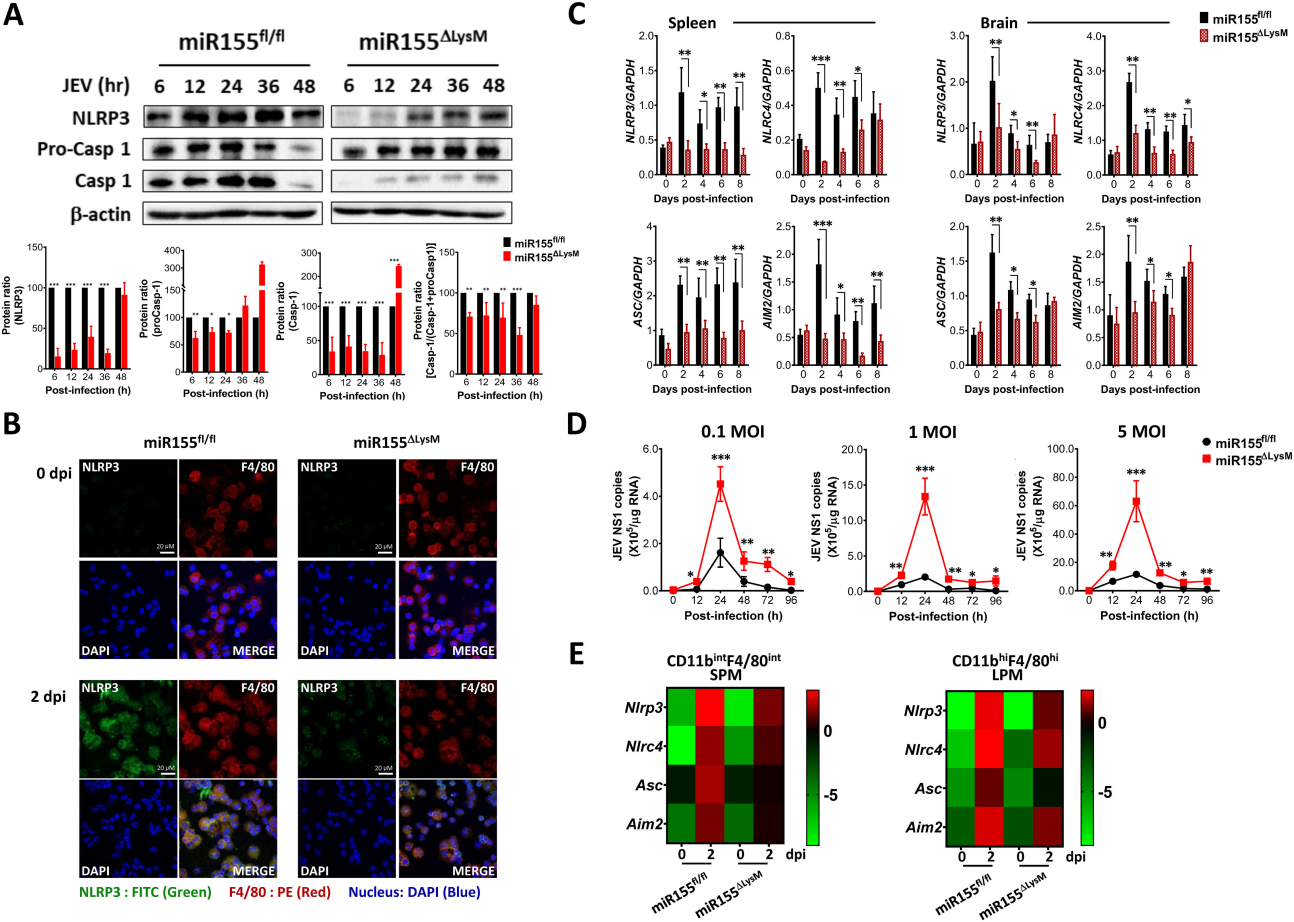
The ablation of miR-155 in macrophages results in impaired activation of NLRP3 inflammasome with facilitated JEV replication. **(A)** Impaired activation of NLRP3 inflammasome in miR155-deificient macrophage. BMDM prepared from miR155^fl/fl^ control and miR155^ΔLysM^ cKO mice was infected with JEV (5 MOI) and used for immunoblotting probed with anti-NLRP3 (110 kDa), anti-pro-caspase-1/caspase-1 (Pro-Casp 1, 45kDa; Caps1, 10 kDa), and anti-β-actin (43 kDa). Bar charts represent the relative levels of NLRP3, Pro–Casp-1, and cleaved Casp-1 normalized to β-actin, as well as the ratio of cleaved Casp-1 to Pro–Casp-1. **(B)** Reduced activation of the NLRP3 inflammasome in macrophages derived from miR155^ΔLysM^ cKO mice during JE progression. Macrophages isolated from the PC of miR155^fl/fl^ control and miR155^ΔLysM^ cKO mice were fixed onto slides and co-stained for NLRP3 (green), F4/80 (red), and the nuclear stain DAPI (blue) for confocal microscopy. **(C)** Reduced expression of inflammasome-related molecules in miR155^ΔLysM^ cKO mice during JE progression. The expression of inflammasome-related molecules was determined by qRT-PCR at the indicated dpi. **(D)** Facilitated replication of JEV in miR155-deficient macrophage. BMDM prepared from miR155^fl/fl^ control and miR155^ΔLysM^ cKO mice was infected with JEV (0.1, 1, 5 MOI, respectively) and JEV replication was assessed by qRT-PCR. Viral RNA load was expressed as viral RNA copy number targeted on JEV NS1 gene per microgram of total RNA. **(E)** Reduced expression of inflammasome-related molecules in macrophages derived from miR155^ΔLysM^ cKO mice during JE progression. Two days after JEV infection, CD11b^int^F4/80^int^ SPMs and CD11b^hi^F4/80^hi^ LPMs sorted from the PC of miR155^fl/fl^ control and miR155^ΔLysM^ cKO mice were used to assess the mRNA expression of inflammasome-related molecules by qRT-PCR. Expression levels were normalized to the housekeeping gene β-actin and presented as the average of at least three independent samples on a log_2_ scale, with colors representing relative expression levels. Data in bar graphs denote the average ± SEM of the levels derived from at least three individual experiments (n=3-4). *p < 0.05; **p < 0.01; ***p < 0.001 between the levels derived from miR155^fl/fl^ control and miR155^ΔLysM^ cKO mice.

The NLR pyrin domain containing 3 (NLRP3) inflammasome is essential in the innate immune response, catalyzing caspase-1 activation that in turn prompts the release of pro-inflammatory cytokines IL-1β and IL-18 in reaction to microbial invasions and cellular damages (50). Notably, the heightened levels of IL-1β in M1 polarized macrophages are closely associated with the activation of NF-κB and MAPK signaling pathways, highlighting the inflammasome’s role in modulating inflammatory responses (51). Our research has uncovered that miR155^ΔLysM^ cKO mice display an impaired polarization towards the M1 phenotype, potentially due to obstructed inflammasome activation. This impairment in M1 macrophage polarization could lead to the worsening of JE, pointing to a significant correlation among inflammasome functionality, M1 macrophage polarization, and the escalation of disease severity, all of which are influenced by the regulation of miR-155. To further elucidate the link between impaired inflammasome function in miR-155-deficient macrophage and JE progression, we examined NLRP3 inflammasome activation in macrophages derived from miR155^ΔLysM^ cKO mice. As depicted in **Figure 6A**, miR155-deficient macrophages exhibit weakened induction of NLRP3 inflammasome activation following JEV infection. This diminished NLRP3 activation caused the inadequate conversion of pro-caspase-1 to its active form caspase-1 in miR-155-deficient macrophages, indicating an impairment of NLRP3 inflammasome activation upon JEV infection. Furthermore, decreased co-localization of NLRP3 and F4/80 antibodies in the peritoneal macrophages from miR155^ΔLysM^ cKO mice corroborates impaired NLRP3 activation, as observed through confocal microscopy (**Figure 6B**). In addition, it was found that miR155^ΔLysM^ cKO mice exhibit lower expression levels of inflammasome-related molecules, such as NLRP3, NLRC4, ASC, and AIM2, in the spleen and brain at the early stage after JEV infection (**Figure 6C**). Unlike M2 macrophages, M1 macrophages engage in the inflammatory response and bolster various antiviral defense mechanisms, including enhanced phagocytosis, to inhibit viral proliferation (21, 22). Hence, the compromised inflammasome activity in miR-155-deficient macrophages leads to weakened M1 macrophage polarization following JEV infection, which may result in a failure to regulate viral proliferation effectively. This scenario was evidenced by the observation that miR-155-deficient macrophages facilitate more rapid and higher levels of viral replication after JEV infection compared to miR155-competent macrophages (**Figure 6D**). Lastly, analyzing inflammasome-related molecules in peritoneal macrophages from miR155^fl/fl^ control and miR155^ΔLysM^ cKO mice post-JEV infection also demonstrated reduced expression of inflammasome activators, including NLRP3, in CD11b^int^F4/80^int^ and CD11b^hi^F4/80^hi^ macrophages purified from the peritoneal cavity of miR155^ΔLysM^ cKO mice (**Figure 6E**). Collectively, these findings indicate that CD11b^int^F4/80^int^ and CD11b^hi^F4/80^hi^ macrophages from miR155^ΔLysM^ cKO mice exhibit impaired NLRP3 inflammasome activation and reduced M1 macrophage polarization upon JEV infection, thereby potentially facilitating increased viral proliferation.

### 3.8 Blocking NLRP3 activation increases the susceptibility to JE

**Figure 7:**
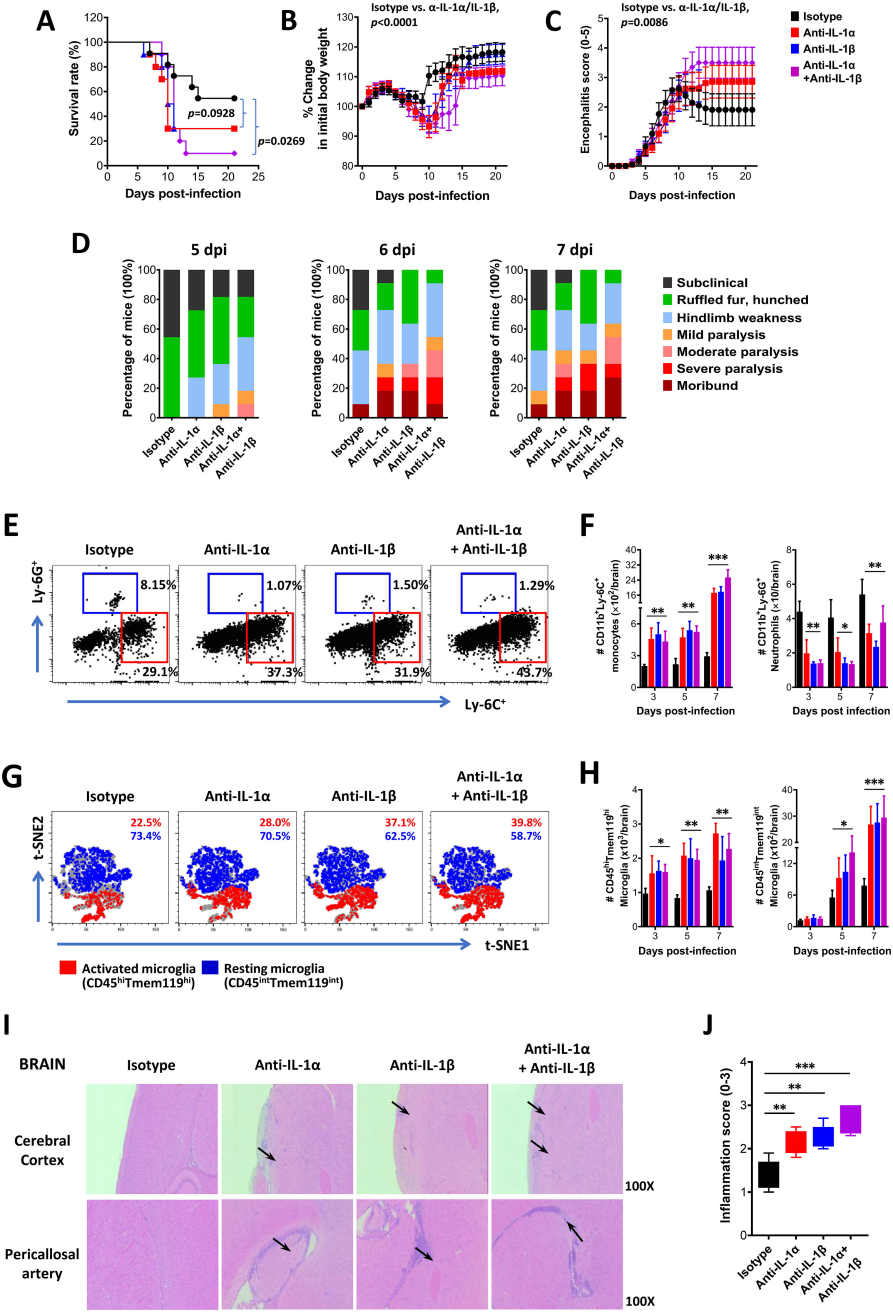
Blocking IL-1α and IL-1β increases vulnerability to JE. **(A–C)** Enhanced susceptibility to JE by treatment of IL-1α and/or IL-1β blocking Abs. Wild-type (WT) mice received i.p. injections of anti-IL-1α (200 μg per mouse), anti-IL-1β (200 μg per mouse), and a cocktail of anti-IL-1α plus anti-IL-1β (each at 200 μg per mouse) 16 h before being infected with JEV. The blocking antibodies were administered again one day post-JEV infection. Following infection, the mice were daily monitored for survival rates **(A)**, changes in body weight **(B)**, and encephalitis severity **(C)**. **(D)** Clinical signs of anti-IL-1α and Il-1β blocking Abs-treated mice. Clinical signs of anti-IL-1α and/or IL-1β blocking Abs-treated mice were monitored and categorized on day 5, 6, and 7 post-infections. **(E, F)** Enhanced CNS infiltration of Ly-6C^+^ monocytes and Ly-6G^+^ neutrophils in mice treated with anti-IL-1α and/or IL-1β blocking antibodies. **(G, H)** Increased accumulation of CD45^hi^Tmem119^hi^ activated microglia in the brains of mice treated with anti-IL-1α and/or IL-1β blocking antibodies. Following thorough cardiac perfusion, the frequency and total number of CD11b^+^Ly-6C^+^ monocytes, CD11b^+^Ly-6G^+^ neutrophils, CD45^hi^Tmem119^hi^ activated microglia, and CD45^int^Tmem119^int^ resting microglia in the brain were analyzed by flow cytometry on day 5 post-infection. Values shown in representative dot plots indicate the average percentages of Ly-6C^+^ monocytes and Ly-6G^+^ neutrophils gated on CD11b^+^ cells. Representative *t*-SNE maps display the average proportions of resting (CD45^int^Tmem119^int^, blue) and activated (CD45^hi^Tmem119^hi^, red) microglia per group. **(I)** Representative photomicrographs of brain sections stained with H&E. Photomicrographs were taken from sagittal sections of the septo-striatal regions of brain derived from WT mice administered with IL-1α and/or IL-1β at 7th dpi. The arrows denote the area of interest. **(J)** Inflammation score of the brain tissues. Inflammation was scored based on the degree of infiltration of inflammatory cells at 7th dpi. Data in bar graphs denote the average ± SEM of the levels derived from at least three individual experiments (n=4-5). *p < 0.05; **p < 0.01; ***p < 0.001 between the levels derived from WT mice treated with anti-IL-1αand/or IL-1β blocking Abs.

Activation of the NLRP3 inflammasome, leading to the secretion of IL-1β and IL-1α, is pivotal in steering macrophages toward an inflammatory M1 phenotype, thereby equipping the immune system to counteract pathogen invasions (52). In miR155^ΔLysM^ cKO mice, a deficiency in NLRP3 activation and the resulting attenuated M1 macrophage polarization were linked to inadequate suppression of early viral replication following JEV infection, aggravating JE progression. This prompted an exploration into the impact of IL-1β and IL-1α, products of NLRP3 inflammasome activation, on the progression of JE. Prior to infecting with JEV, we administered neutralizing Abs against IL-1α and IL-1β and subsequently monitored the mortality and morbidity of the infected mice on a daily basis. The results indicated that WT mice treated with neutralizing Abs against IL-1α and/or IL-1β exhibited increased susceptibility to JE compared to those injected with isotype controls (**Figure 7A**). Mice injected with isotype controls demonstrated approximately 40% mortality, whereas those treated with neutralizing Abs against IL-1α and/or IL-1β showed significantly higher mortality rates, with reaching around 70% mortality in both groups. Moreover, mice that received simultaneous injections of neutralizing Abs against both IL-1α and IL-1β exhibited an even higher mortality rate of approximately 90%. In addition, mice treated with neutralizing Abs against IL-1α and/or IL-1β experienced more pronounced weight loss between days 7 and 15 post-infection (**Figure 7B**), and higher encephalitis scores were recorded in these mice, indicating a more severe JE progression (**Figure 7C**). To further clarify the clinical manifestations of JE in mice treated with anti-IL-1α and/or anti-IL-1β neutralizing Abs, we monitored the clinical signs of infected mice, focused on seven specific indicators in JE progression. As expected, mice treated with anti-IL-1α and/or anti-IL-1β neutralizing Abs exhibited severe clinical signs from 5 to 7 dpi, compared to those treated with isotype control (**Figure 7D**). The most severe clinical signs were observed in the group treated with both anti-IL-1α and IL-1β neutralizing Abs. Furthermore, given the impaired NLRP3 activation in miR155-deficient macrophages, we investigated the alterations in susceptibility to JE in response to administration of NLRP3-specific inhibitor MCC950. Even though the administration of MCC950 induced a somewhat milder change compared to treatment with anti-IL-1α and IL-1β neutralizing Abs, it significantly increased susceptibility to JE progression (**Supplementary Figure 5A**). Mice treated with a vehicle exhibited approximately 40% mortality, whereas those administered MCC950 displayed around 70% mortality. Mice receiving MCC950 also showed greater weight loss between 7 and 15 dpi compared to the vehicle-treated group (**Supplementary Figure 5B**), with a correspondingly higher encephalitis score (**Supplementary Figure 5C**). Similarly, mice treated with MCC950 exhibited more severe clinical symptoms on 5, 6, and 7 dpi (**Supplementary Figure 5D**). These findings suggest that both the neutralization of the inflammasome effector molecules IL-1α and IL-1β and the inhibition of NLRP3 by MCC950 increase susceptibility to JE progression induced by JEV infection.

To better understand the increased susceptibility to JE following administration of IL-1α and/or IL-1β neutralizing antibodies, we analyzed the infiltration of Ly-6C^+^ monocytes and Ly-6G^+^ neutrophils into the CNS tissue (brain). Our results revealed an increased presence of Ly-6C^+^ monocytes—but not Ly-6G^+^ neutrophils—in mice treated with IL-1α and/or IL-1β neutralizing antibodies compared to those administered isotype control antibodies (**Figure 7E**). Consequently, there was a significant increase in the total number of infiltrating Ly-6C^+^ monocytes in the brains of treated mice (**Figure 7F**). Furthermore, assessment of microglial activation showed that mice treated with IL-1α and/or IL-1β neutralizing Abs exhibited a higher proportion and overall number of activated microglia compared to resting microglia (**Figures 7G, H**). Lastly, a histopathological examination of the brain was performed following the administration of IL-1α and/or IL-1β neutralizing Abs. As anticipated, mice treated with these neutralizing Abs after JEV infection exhibited more pronounced infiltration of inflammatory cells around cerebral blood vessels (**Figure 7I**). Consequently, the anti–IL-1α and/or IL-1β Abs-treated mice showed higher brain inflammation scores (**Figure 7J**). Therefore, these results indicate that administration of IL-1α and IL-1β neutralizing Abs exacerbates CNS neuroinflammation during the course of JE following JEV infection.

### 3.9 Blocking IL-1α and IL-1β impairs M1 polarization of macrophages in JE progression

**Figure 8:**
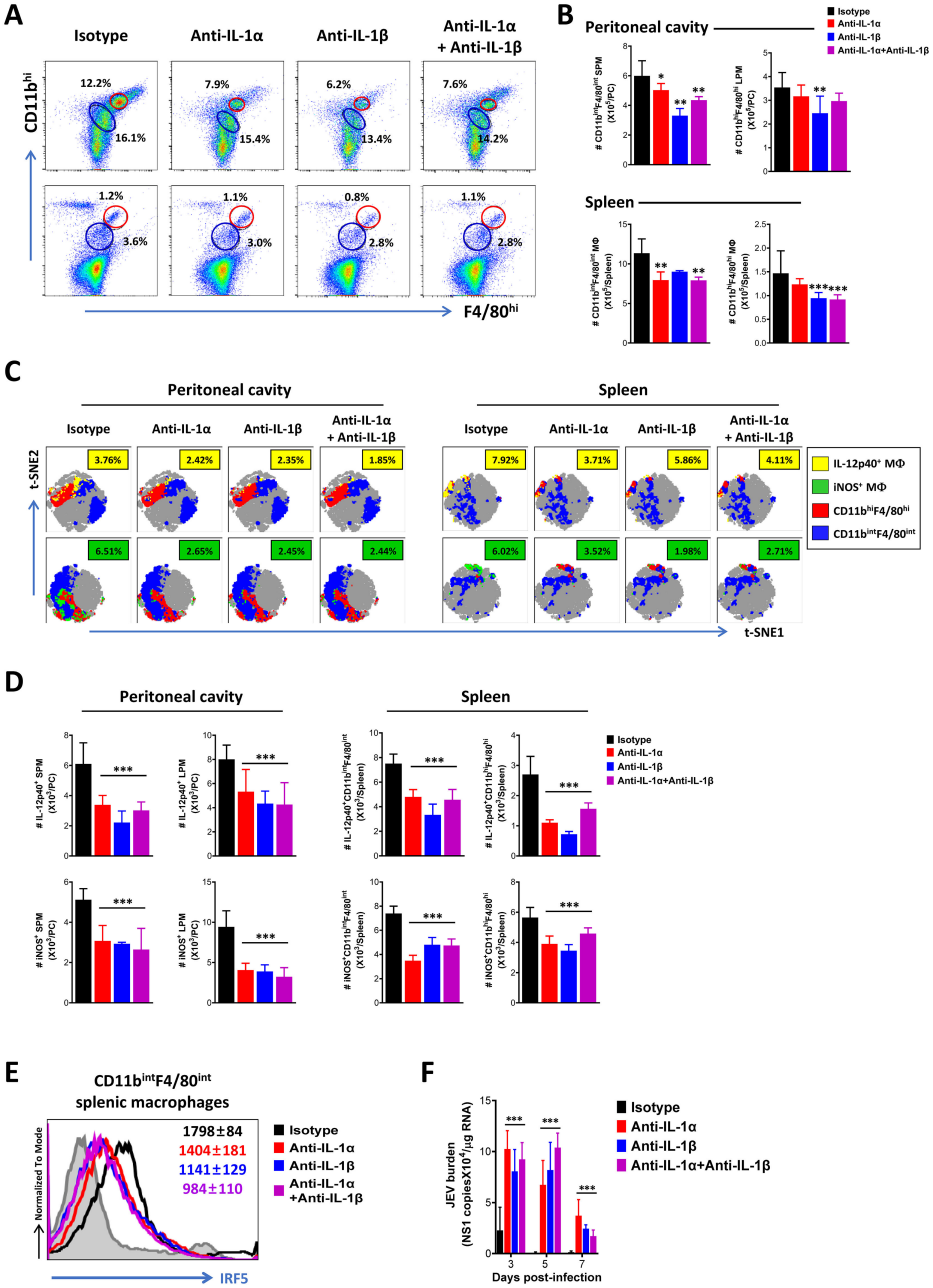
Blocking IL-1α and/or IL-1β suppresses M1 macrophage polarization during JE progression. **(A)** Representative flow cytometric analysis of the CD11b^+^F4/80^+^ macrophage subpopulations in the peritoneal cavity (PC) and spleen of mice treated with IL-1α and/or IL-1β blocking Abs following JEV infection. **(B)** Quantification of CD11b^int^F4/80^int^ and CD11b^hi^F4/80^hi^ macrophages in the PC and spleen. Both frequency and total number of these macrophage subsets were assessed by flow cytometry at 2 dpi. Representative dot plots show the percentages of each subset. **(C)** *t*-SNE plots illustrating the distribution of IL-12p40^+^ and iNOS^+^ M1 macrophages in the PC and spleen. These maps display the average frequencies of IL-12p40 and iNOS-producing macrophages per group at 2 dpi. **(D)** Total number of IL-12p40^+^ and iNOS^+^ CD11b^int^F4/80^int^ and CD11b^hi^F4/80^hi^ macrophages in the PC and spleen. Cells were briefly stimulated with LPS *ex vivo*, followed by intracellular cytokine staining in combination with CD11b and F4/80 surface markers to determine cytokine production levels. **(E)** IRF5 expression in CD11b^int^F4/80^int^ splenic macrophages. The mean fluorescence intensity (MFI) of IRF5 was quantified using intracellular staining in macrophages isolated from the spleens of IL-1α and/or IL-1β blocking Ab-treated mice at 2 dpi. Histograms show the average ± SEM of IRF5 MFI. **(F)** JEV viral burden in extraneural tissue (spleen) at early stages post-infection. Viral RNA load in the spleen was quantified by qRT-PCR targeting the JEV NS1 gene and expressed as the number of viral RNA copies per microgram of total RNA at 3, 5, 7 dpi. Bar graphs represent the mean ± SEM from at least three independent experiments (n = 3-4). *p < 0.05; **p < 0.01; ***p < 0.001, compared to wild-type (WT) mice treated with anti-IL-1α and/or IL-1β blocking Abs.

Finally, to elucidate the association between increased susceptibility to JE following administration of IL-1α and IL-1β neutralizing Abs and M1 macrophage polarization, we analyzed the M1 polarization of peritoneal and splenic macrophages in the early stages post-JEV infection. It was observed that mice injected with IL-1α and/or IL-1β neutralizing Abs exhibited a reduction in the distribution of CD11b^int^F4/80^int^ SPM in the peritoneum and CD11b^int^F4/80^int^ macrophages in the spleen after JEV infection (**Figure 8A**). Consequently, there was a significant decrease in the total number of CD11b^int^F4/80^int^ SPM and CD11b^int^F4/80^int^ macrophages present in both the peritoneum and spleen (**Figure 8B**). However, no significant change was observed in the peritoneal CD11b^hi^F4/80^hi^ LPM and splenic CD11b^hi^F4/80^hi^ macrophages. Further investigation into IL-12p40 and iNOS-producing M1 macrophages revealed a lower detection of IL-12p40 and iNOS-producing M1 macrophages in both the peritoneum and spleen of IL-1α and/or IL-1β neutralizing Abs-injected mice at the early stage after JEV infection (**Figure 8C**). This led to a reduced overall count of IL-12p40 and iNOS-producing CD11b^int^F4/80^int^ and CD11b^hi^F4/80^hi^ macrophages in the peritoneum and spleen of IL-1α and IL-1β neutralizing Abs-injected mice (**Figure 8D**). In addition, mice treated with IL-1α and/or IL-1β neutralizing Abs exhibited lower expression of IRF5, transcription factor for M1 macrophages, in the CD11b^int^F4/80^int^ splenic macrophages compared to those treated with isotype control (**Figure 8E**). Also, our results revealed that this impaired M1 macrophage polarization in anti-IL-1α and/or IL-1β Abs-treated mice appeared to increase the viral load within the spleen in the early stages after JEV infection (**Figure 8F**), potentially leading to diminished suppression of viral proliferation at the extraneural peripheral sites. Collectively, these results suggest that administration of IL-1α and IL-1β neutralizing Abs suppresses M1 macrophage polarization at the early stage after JEV infection, thereby enhancing viral proliferation in peripheral tissues. This enhanced viral proliferation is likely to increase the viral load invading the CNS tissues, thereby elevating susceptibility to JE.

## 4 Discussion

The elevation of miR-155 in the serum of JE patients suggests its potential involvement in JE pathogenesis (34). CNS macrophages, notably microglia, are found to upregulate miR-155 expression following JEV infection (35), which is postulated to contribute to inhibiting viral replication. Despite these indications, there is a scarcity of empirical evidence elucidating the role of miR155 in specific immune cells during JE progression using practical infection models. In the present study, we propose that only mice deficient in myeloid cell-specific miR-155 expression (miR155^ΔLysM^ cKO mice) are more significantly susceptible to JE progression. Following JEV infection, miR155^ΔLysM^ cKO mice exhibited heightened Ly-6C^+^ monocyte infiltration from peripheral blood to the CNS, and an increased prevalence of CD45^hi^Tmem119^hi^ activated microglia, concomitant with augmented viral burdens in both spleen and CNS. miR155^ΔLysM^ cKO mice displayed a compromised IFN-I response and a diminished innate cytokine response in the spleen in the early stage of infection, which transitioned to a surge in pro-inflammatory cytokine expression in the CNS during later stages. Concurrently, these mice demonstrated decreased accumulation of IL-12p40 and iNOS-producing M1 macrophages early in JEV infection, followed by an attenuated JEV-specific CD4^+^ and CD8^+^ T-cell responses as JE advanced. The diminished M1 macrophage accumulation in miR155^ΔLysM^ cKO mice correlated with a downregulation of NLRP3 inflammasome activation. This correlation was further evidenced by the increased susceptibility to JE and decreased M1 macrophage accumulation upon administration of either the NLRP3 inflammasome inhibitor MCC950 or IL-1α and IL-1β blocking Abs. Collectively, these findings imply that a deficit in miR-155 within macrophages leads to a reduction of NLRP3 inflammasome activation during the early stage of JEV infection, culminating in impaired M1 macrophage polarization. This impairment potentially facilitates the failure of viral clearance in peripheral tissues at the early stage of infection, thereby exacerbating neuroinflammation in the CNS as JE progresses.

miR-155 appears to play diverse roles in the context of various diseases. miR-155 enhances lung injury in endotoxemic mice by increasing CCL2 production from macrophages (53), and exosomal miRNA-155-5p from M1 macrophages targets GDF6 to inhibit angiogenesis, thereby impeding diabetic wound healing (54). Conversely, miR-155-deficient mice exhibit attenuated colitis (55), and administration of miR-155 antagomir suppresses DSS-induced colitis by targeting Jarid/Wnt-β-catenin to modulate the Th17/Treg balance (56). Furthermore, miR-155 exhibits both pro-atherosclerotic and anti-atherosclerotic functions (57), indicating its diverse effects on disease pathogenesis. In particular, noteworthy is the distinct roles of miR-155 depending on cell type and disease context. As strikingly exemplified in the EAE model, T cell-specific deficiency of miR-155 suppresses CNS neuroinflammation, whereas macrophage-specific ablation of miR-155 expression using LysM-Cre does not impact EAE development, despite high miR-155 expression in infiltrated macrophages during EAE (58). Considering monocytes/macrophages as primary target cells for viral replication following JEV infection, miR155^ΔLysM^ cKO mice, as proposed by our study, may exhibit increased susceptibility to JE. Conversely, miR155^ΔCD4^ and miR155^ΔCD19^ cKO mice, generated using CD4-Cre and CD19-Cre, respectively, exhibited no significant changes in susceptibility to JE. In contrast, miR155^ΔCD11c^ cKO mice, generated using CD11c-Cre, displayed a modest increase in susceptibility, accompanied by a suppressed T cell response. However, this effect was less pronounced compared to that observed in miR155^ΔLysM^ cKO mice. Ultimately, these findings highlight the significant contribution of myeloid cells, including monocytes/macrophages, to JE progression and underscore the critical importance of miR-155 expression in myeloid cells for resistance against JE progression.

Typically, miR-155 is believed to be involved in promoting inflammatory responses, contrasting with the proposed role of miR-146a (59). miR-155 is considered an NF-κB-dependent miRNA, with its expression induced in macrophages by TLR stimulation, alongside with miR-146a. The induction of miR-155 expression upon TLR stimulation has been observed through aberrant TLR activation induced by microbial pathogen infections. For instance, infection of human PBMCs with *Francisella tularensis* induces miR-155 expression via downregulation of TLR-dependent SHIP1 (60), and Leishmania RNA virus 1 induces miR-155 expression in murine macrophages via TLR3 activation (61). In addition, HMGB-1 regulates miR-155 expression in a TLR2/MyD88-dependent manner (62). Similarly, microglial infection by JEV induces miR-155 expression, contributing to the suppression of virus proliferation through the promotion of innate immune gene expression (35). Ultimately, these observations suggest that monocytes/macrophages infected with JEV induce miR-155 expression via TLR stimulation (16), thereby contributing to the manifestation of resistance against JE through the induction of innate immune responses mediated by miR-155. Consistently, in our current study, miR155^ΔLysM^ cKO mice exhibited impaired innate immune responses during the early stages of JEV infection. Furthermore, strongly supporting our findings, mice infected with WNV showed a significant increase in miR-155 expression in the brain (63), and subsequently miR-155-deficient mice displayed more severe neurological symptoms following lethal and non-lethal WNV infections (64). Also, miR-155 overexpression in neuronal cells suppressed WNV proliferation. However, the identification of the cell type responsible for inducing susceptibility to WNV infection using miR-155-deficient mice remains elusive. Given that WNV, like JEV, induces viral encephalitis through similar pathogenesis as a neurotrophic virus, it can be inferred that miR-155 expression in macrophages is crucial during WNV infection. These discussions ultimately underscore the significant role of miR-155 expression in macrophages after neurotrophic virus infections, including JEV, in inhibiting viral encephalitis.

One intriguing finding in this study is that the deficiency of miR155 in macrophages leads to increased susceptibility to JE due to impaired M1 macrophage polarization. Furthermore, we found that impaired NLRP3 inflammasome activation in macrophages lacking miR-155 was associated with suppressed M1 polarization. Indeed, according to several reports, miR-155 in macrophages is thought to be linked to their M1 polarization. For instance, miR-155 deletion has been shown to promote alternative M2 macrophage polarization, thereby enhancing resistance to colitis (55), and the involvement of SOCS1/miR-155 axis in M1 macrophage polarization during *Staphylococcus aureus*-induced respiratory infection has been described (65). However, the detailed pathway by which miR-155 expression induces M1 macrophage polarization remains elusive. In our current study, we proposed that miR-155-deficient macrophages exhibited impaired NLRP3 inflammasome activation, which subsequently hindered M1 macrophage polarization. This scenario is strengthened by the results indicating that miR-155 enhances NLRP3 inflammasome activation via the ERK1/2 pathway in macrophages (66). Furthermore, miR-155 upregulation appears to activate non-canonical inflammasome activation and promote *Porphyromonas gingivalis*-induced macrophage pyroptosis through the regulation of NLRP3 inflammasome (67). These findings suggest that miR-155 may act as a key regulator of NLRP3 inflammasome activation. However, our study did not elucidate the detailed pathway through which miR-155 deficiency induces impaired NLRP3 inflammasome activation. Comprehensive analysis of miR-155 candidate target gene expression indicates that Peli1, Jarid2, and Bcl6 may contribute, either directly or indirectly, to the impaired NLRP3 inflammasome activation and reduced M1 macrophage polarization observed in miR-155-deficient conditions (68–70). Furthermore, miR-155 may influence key components of TLR signaling pathways, including TLR4 and TLR3 (16, 60–62), as well as modulators such as ERK1/2 and SOCS1 (33, 66, 71). Nonetheless, a notable result of our study is that the administration of the NLRP3 inflammasome inhibitor MCC950 and blocking Abs of effector cytokines IL-1α and IL-1β induced increased susceptibility to JE. Particularly, the inhibition of M1 macrophage polarization was observed upon administration of IL-1α and IL-1β blocking Abs. These results support the notion that the effector cytokines IL-1α and IL-1β of NLRP3 inflammasome activation contribute to M1 macrophage polarization. In particular, several lines of evidence suggest that IL-1β promotes M1 macrophage polarization. IL-1β suppresses M2 macrophage polarization through CD220 downregulation (72), and inhibition of histone crotonylation mediated by acyl-CoA synthetase short-chain family member 2 (ACSS2) alleviates IL-1β-dependent macrophage activation (73). Additionally, IL-1β-activated M1 macrophages promote myocardial fibrosis in diabetes (74). Conversely, macrophages serve as the major producers of IL-1α and IL-1β, thereby promoting tumorigenesis through paracrine AIM2 inflammasome activation in neighboring macrophages (75), and arteriogenesis through autocrine STAT3/NF-kB-mediated transcription of pro-angiogenic VEGF-A in macrophages (76). Thus, these notions collectively suggest that NLRP3 inflammasome activation induced by miR-155 in primary target cell macrophages during JEV infection stimulates the production of IL-1α and IL-1β, which in turn promote M1 macrophage polarization via autocrine and paracrine mechanisms, whereas miR-155 deficiency in macrophages may lead to increased susceptibility to JE by impairing these processes.

In our study, we underscore the significance of M1 macrophage polarization and NLRP3 inflammasome activation mediated by IL-1α and IL-1β in JE progression following JEV infection. The increase in M1 macrophages, particularly in immunologically privileged sites like the brain, would rather facilitate CNS neuroinflammation through uncontrolled production of inflammatory cytokines (40). Consequently, our findings revealed no significant difference in the distribution of M1 macrophages accumulated in the brain between miR155^fl/fl^ control and miR155^ΔLysM^ cKO mice; instead, a phenomenon was observed where a greater absolute total number of IL-12p40 and iNOS-producing M1 macrophages accumulated within the brain of miR155^ΔLysM^ cKO mice. This latter phenomenon is presumed to be induced by increased inflammatory responses within the brain of miR155^ΔLysM^ cKO mice at later stages after JEV infection Therefore, the manifestation of resistance to JE progression through increased accumulation of M1 macrophages is anticipated to be facilitated by viral clearance in peripheral lymphoid tissue or extraneural tissue at the early stage. In support, our experimental results showed the rapid replication of JEV in miR-155-deficient macrophages after JEV infection, which suggests that impaired M1 macrophage polarization ultimately leads to increased virus load invading CNS tissue, due to the failure of viral clearance in peripheral tissue at the initial stages of infection. In addition, our previous studies have elucidated the critical role of M1 macrophage accumulation in extraneural tissues during the early stages of JEV infection in suppressing JE progression (38). Furthermore, it is believed that IL-1β produced by increased M1 macrophages post-JEV infection may inhibit JEV replication through synergistic interactions with IFN-I proteins (77). Therefore, in conclusion, we emphasize the importance of promoting rapid differentiation of M1 macrophages in extraneural tissue during the early stages of JEV infection to reduce virus load invading CNS tissue, thereby manifesting resistance to JE. In this series of processes, it is proposed that miR-155 expressed in macrophages plays a crucial role as a key regulator of NLRP3 inflammasome activation and M1 macrophage polarization in suppressing JE progression.

## Data Availability

The original contributions presented in the study are included in the article/**Supplementary Material**. Further inquiries can be directed to the corresponding author.
