# Supplementary material for: Myeloid miR-155 deficiency exacerbates viral encephalitis by hindering M1 macrophage polarization due to impaired NLRP3 inflammasome activation in extraneural tissues
Source: Front Immunol. 2026 Jun 11;17:1818106. doi: 10.3389/fimmu.2026.1818106 (PMC13294391; doi:10.3389/fimmu.2026.1818106)
Supplement: Supplementary file 1 [file DataSheet1.pdf]

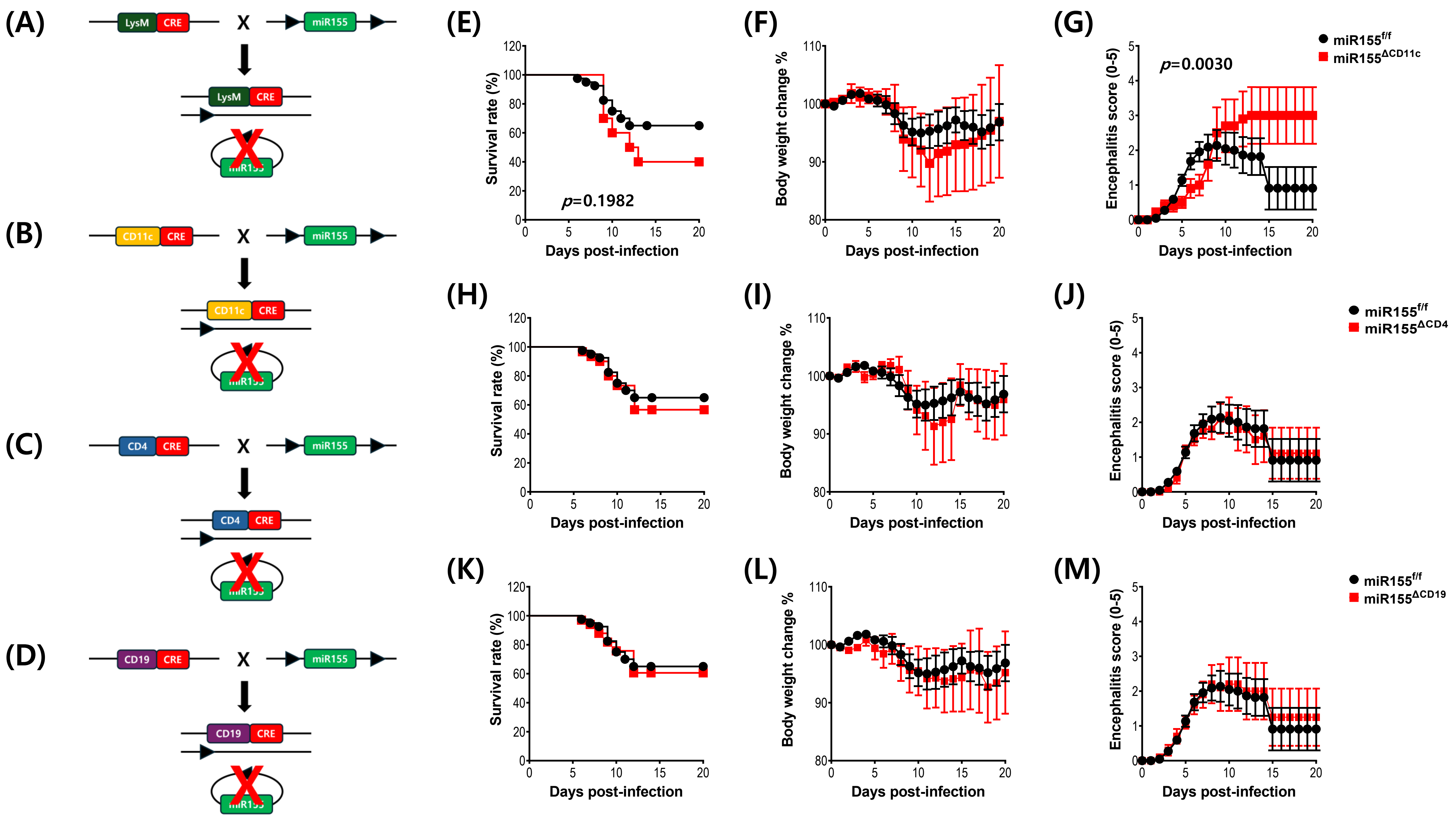

(A)
LysM
CRE
X
miR155
(B)
CD11c
CRE
(C)
CD4
CRE
(D)
CD19
CRE
(E)
Survival rate (%)
Days post-infection
p=0.1982
(F)
Body weight change %
Days post-infection
(G)
Encephalitis score (0-5)
Days post-infection
p=0.0030
miR155f/f
miR155ΔCD11c
(H)
Survival rate (%)
Days post-infection
(I)
Body weight change %
Days post-infection
(J)
Encephalitis score (0-5)
Days post-infection
miR155f/f
miR155ΔCD4
(K)
Survival rate (%)
Days post-infection
(L)
Body weight change %
Days post-infection
(M)
Encephalitis score (0-5)
Days post-infection
miR155f/f
miR155ΔCD19

Figure S1. The expression of miR-155 in myeloid cells plays a critical role in suppressing the progression of JE. (A-D) Scheme for the generation of specific deletion of miR155 in immune cells. miR155$^{\Delta CD4}$, miR155$^{\Delta CD19}$, miR155$^{\Delta CD11c}$ and miR155$^{\Delta LysM}$ cKO mice were generated by crossbreeding miR155$^{fl/fl}$ mice with CD4-Cre, CD19-Cre, CD11c-Cre, and LysM Cre, respectively, using the Cre-LoxP recombination system. (E-M) JE Susceptibility in miR-155 cKO mice. miR155$^{\Delta CD4}$, miR155$^{\Delta CD19}$, and miR155$^{\Delta CD11c}$ cKO mice were infected with JEV (2.5×10^7 FFU) via i.p. route, alongside with miR155$^{fl/fl}$ control mice. Infected mice were monitored over 20 days. (E-G) Susceptibility of miR155$^{fl/fl}$ and miR155$^{\Delta CD11c}$ cKO mice to JE progression. (H-J) Susceptibility of miR155$^{fl/fl}$ and miR155$^{\Delta CD4}$ cKO mice to JE progression. (K-M) Susceptibility of miR155$^{fl/fl}$ and miR155$^{\Delta CD19}$ cKO mice to JE progression. (E, H, K) The proportion of surviving mice in each group was monitored daily for 20 days. (F, I, L) Changes in body weight. Data is expressed as the average ± SEM of body weight relative to the time of challenge. (G, J, M) Encephalitis score. Mice infected with JEV were expressed as the average score ± SEM of each group.
