# Supplementary material for: Myeloid miR-155 deficiency exacerbates viral encephalitis by hindering M1 macrophage polarization due to impaired NLRP3 inflammasome activation in extraneural tissues
Source: Front Immunol. 2026 Jun 11;17:1818106. doi: 10.3389/fimmu.2026.1818106 (PMC13294391; doi:10.3389/fimmu.2026.1818106)
Supplement: Supplementary file 2 [file DataSheet2.pdf]

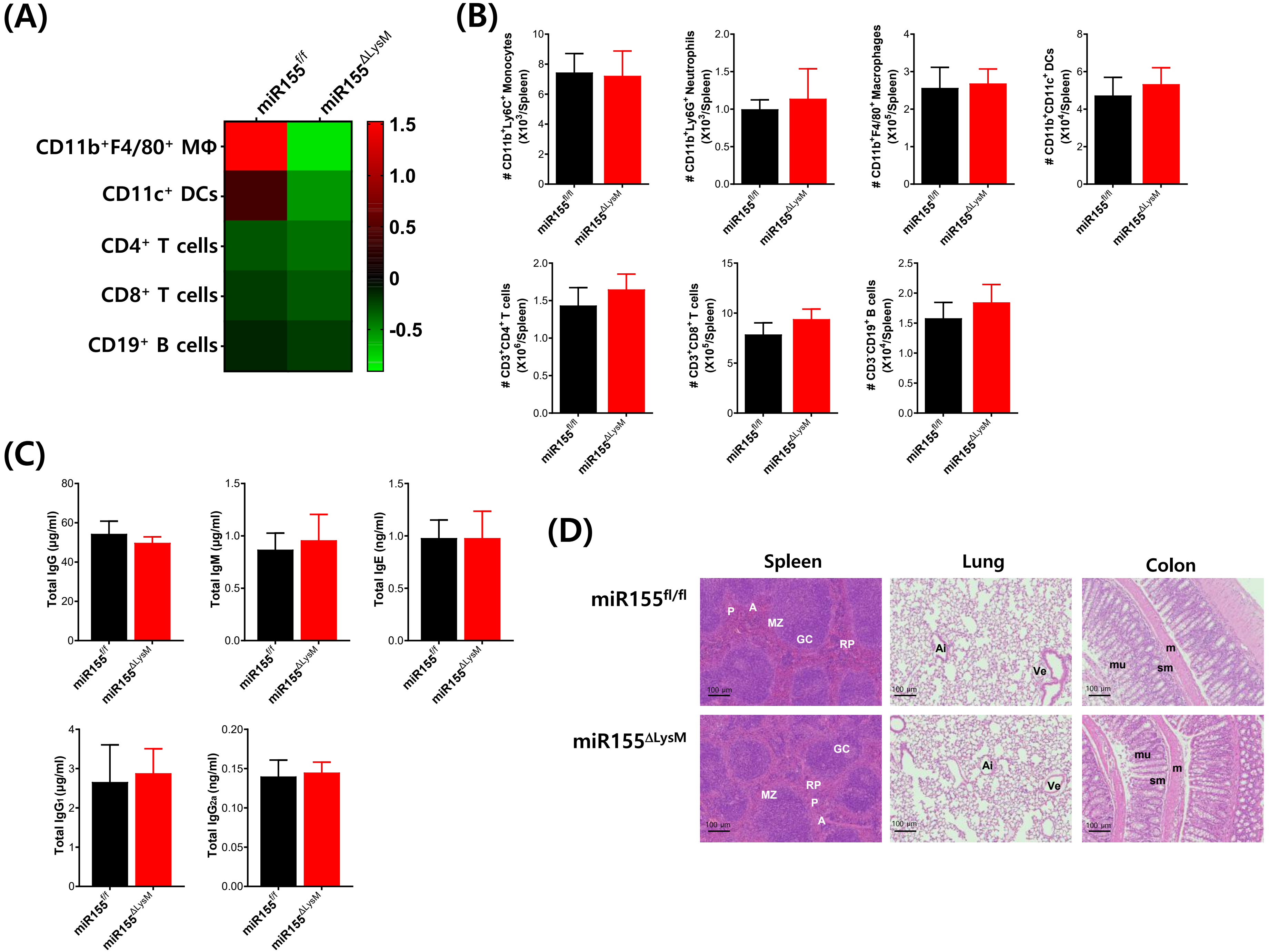

(A)
miR155f/f
miR155ΔLysM
CD11b+F4/80+ MΦ
CD11c+ DCs
CD4+ T cells
CD8+ T cells
CD19+ B cells
1.5
1.0
0.5
0
-0.5
(B)
CD11b+Ly6C+ Monocytes (X10^3/Spleen)
CD11b+Ly6G+ Neutrophils (X10^3/Spleen)
CD11b+F4/80+ Macrophages (X10^5/Spleen)
CD11b+CD11c+ DCs (X10^4/Spleen)
CD3+CD4+ T cells (X10^6/Spleen)
CD3+CD8+ T cells (X10^5/Spleen)
CD3-CD19+ B cells (X10^4/Spleen)
miR155fl/fl
miR155ΔLysM
(C)
Total IgG (μg/ml)
Total IgM (μg/ml)
Total IgE (ng/ml)
Total IgG1 (μg/ml)
Total IgG2a (ng/ml)
miR155f/f
miR155ΔLysM
(D)
Spleen
Lung
Colon
miR155fl/fl
miR155ΔLysM
P
A
MZ
GC
RP
Ai
Ve
mu
m
sm
100 μm

Figure S2. miR-155 expression and immune phenotypes of LysM-Cre miR155$^{fl/fl}$ (miR155$^{\Delta LysM}$) cKO mice. (A) miR-155 expression in myeloid and lymphoid cells. miR-155 levels in myeloid and lymphoid cells were assessed by real-time qRT-PCR using microRNAs extracted from sorted cell populations of miR155$^{fl/fl}$ and miR155$^{\Delta LysM}$ cKO mice. Expression levels were normalized to control snoRNA142 and are presented as the average of at least four independent samples, shown in the indicated colors on a $\log_2$ scale. (B) Immune phenotypic analysis of miR155$^{\Delta LysM}$ cKO mice in the spleen. The total number of immune cell subsets (CD4$^+$ T cells, CD8$^+$ T cells, CD19$^+$ B cells, Ly-6C$^+$ monocytes, Ly-6G$^+$ neutrophils, CD11b$^+$F4/80$^+$ macrophages, and CD11c$^+$ conventional dendritic cells) was quantified by flow cytometry. (C) Serum immunoglobulin isotype levels. The levels of serum immunoglobulin isotypes were measured using conventional ELISA. (D) Histological examination of spleen, lung, and colon tissues. Representative histological sections are shown. Data represent the mean ± SEM from at least two independent experiments (n = 3–4). p<0.05, p<0.01, p<0.001, miR155$^{fl/fl}$ vs. miR155$^{\Delta LysM}$ cKO mice. mu, mucosa; sm, submucosa; m, musculosa; A, central artery; Ai, airway; GC, germinal center; MZ, marginal zone; P, periarterial lymphatic sheath (PALS); RP, red pulp; Ve, vessel.
