# Supplementary material for: Myeloid miR-155 deficiency exacerbates viral encephalitis by hindering M1 macrophage polarization due to impaired NLRP3 inflammasome activation in extraneural tissues
Source: Front Immunol. 2026 Jun 11;17:1818106. doi: 10.3389/fimmu.2026.1818106 (PMC13294391; doi:10.3389/fimmu.2026.1818106)
Supplement: Supplementary file 3 [file DataSheet3.pdf]

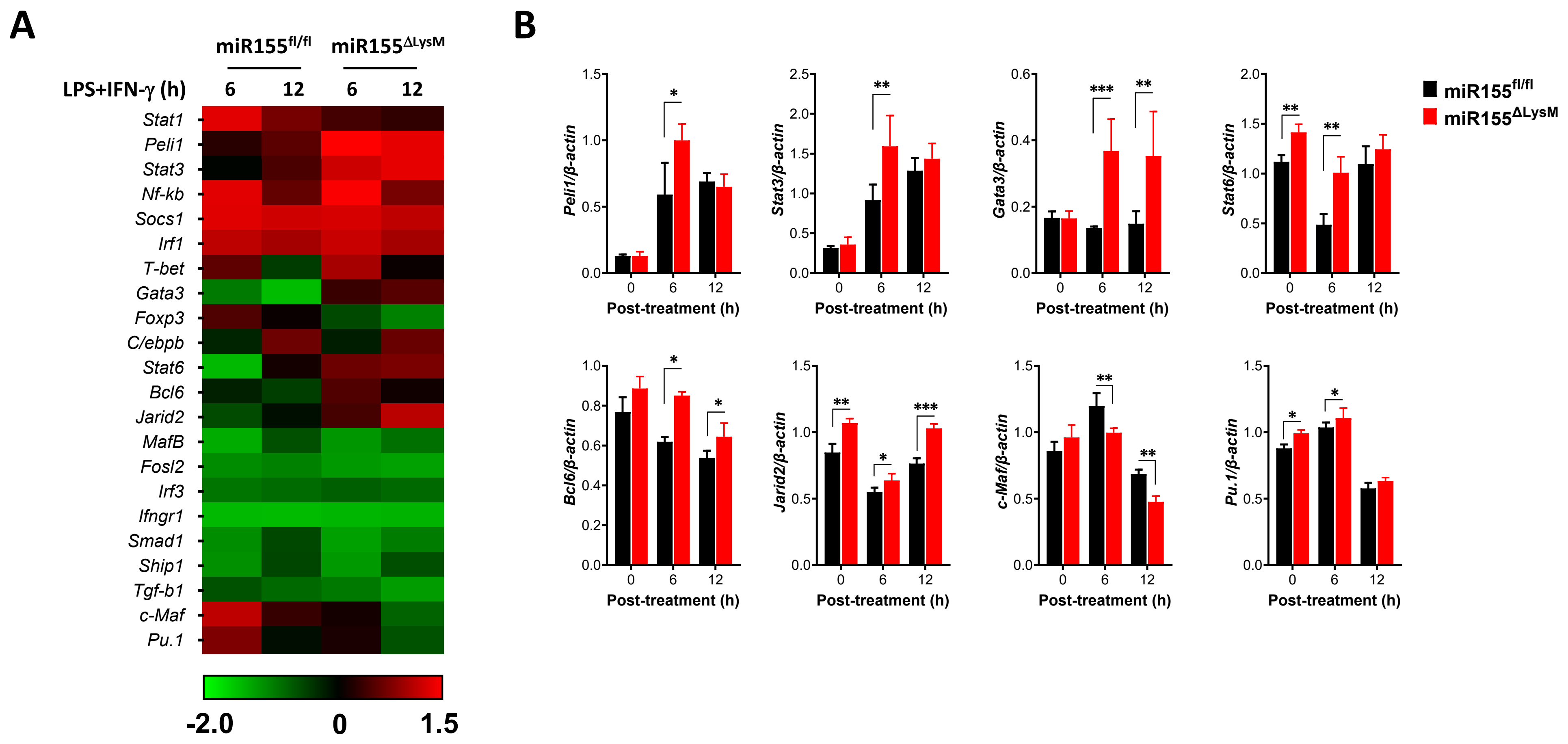

Figure S3. Transcription factors regulated by miR-155 in macrophages during M1 polarization in response to LPS and IFN-γ stimulation. BMDM from miR155$^{fl/fl}$ control and miR155$^{\Delta LysM}$ cKO mice were stimulated with LPS and IFN-γ for 6 and 12 h. (A) Heatmap showing the expression of miR-155-targeted transcription factors in LPS+IFN-γ-stimulated BMDM. Expression levels of transcription factors were normalized to their respective levels in unstimulated BMDM, following normalization with the housekeeping gene β-actin. Data are displayed as the average of four independent samples on a $\log_2$ scale, with colors representing relative expression levels. (B) Validation of the expression levels of selected transcription factors in macrophages following LPS and IFN-γ stimulation. Bar graphs represent the mean ± SEM of values derived from at least two independent experiments (n=4–5). Statistical significance is indicated as *$p<0.05$, **$p<0.01$, and ***$p<0.001$, comparing BMDMs from miR155$^{fl/fl}$ control and miR155$^{\Delta LysM}$ cKO mice.
