# Supplementary material for: Myeloid miR-155 deficiency exacerbates viral encephalitis by hindering M1 macrophage polarization due to impaired NLRP3 inflammasome activation in extraneural tissues
Source: Front Immunol. 2026 Jun 11;17:1818106. doi: 10.3389/fimmu.2026.1818106 (PMC13294391; doi:10.3389/fimmu.2026.1818106)
Supplement: Supplementary file 4 [file DataSheet4.pdf]

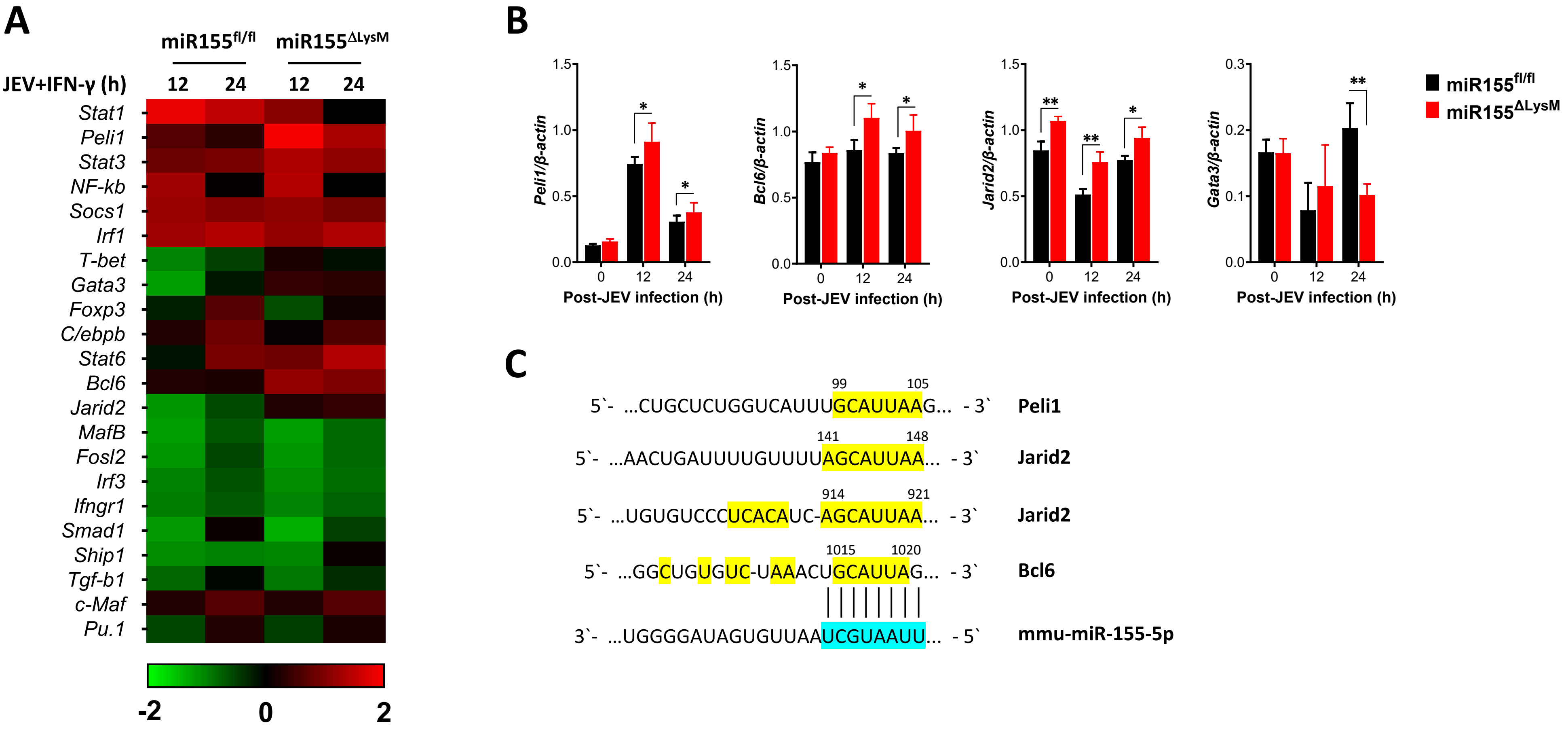

Figure S4. Transcription factors regulated by miR-155 in macrophages during M1 polarization in response to JEV infection. BMDM from miR155$^{fl/fl}$ control and miR155$^{\Delta LysM}$ cKO mice were infected with JEV for 12 and 24 hours. (A) Heatmap illustrating the expression of miR-155-targeted transcription factors in JEV-infected BMDMs. Expression levels were normalized to those in uninfected BMDMs, with additional normalization using the housekeeping gene β-actin. Data are presented as the average of four independent samples on a $\log_2$ scale, with colors representing relative expression levels. (B) Validation of the expression levels of selected transcription factors in macrophages following JEV infection. (C) Alignment of predicted miR-155 target sites within the 3'-UTR regions of transcription factors showing altered expression. Predicted miR-155 target sites were analyzed using miRNA alignment tools (TargetScan: www.targetscan.org; miRDB: www.mirdb.org). Bar graphs represent the mean ± SEM of data obtained from at least two independent experiments (n=4–5). Statistical significance is denoted as *p<0.05, **p<0.01, and ***p<0.001, comparing BMDMs from miR155$^{fl/fl}$ control and miR155$^{\Delta LysM}$ cKO mice.
