# Supplementary material for: Myeloid miR-155 deficiency exacerbates viral encephalitis by hindering M1 macrophage polarization due to impaired NLRP3 inflammasome activation in extraneural tissues
Source: Front Immunol. 2026 Jun 11;17:1818106. doi: 10.3389/fimmu.2026.1818106 (PMC13294391; doi:10.3389/fimmu.2026.1818106)
Supplement: Supplementary file 5 [file DataSheet5.pdf]

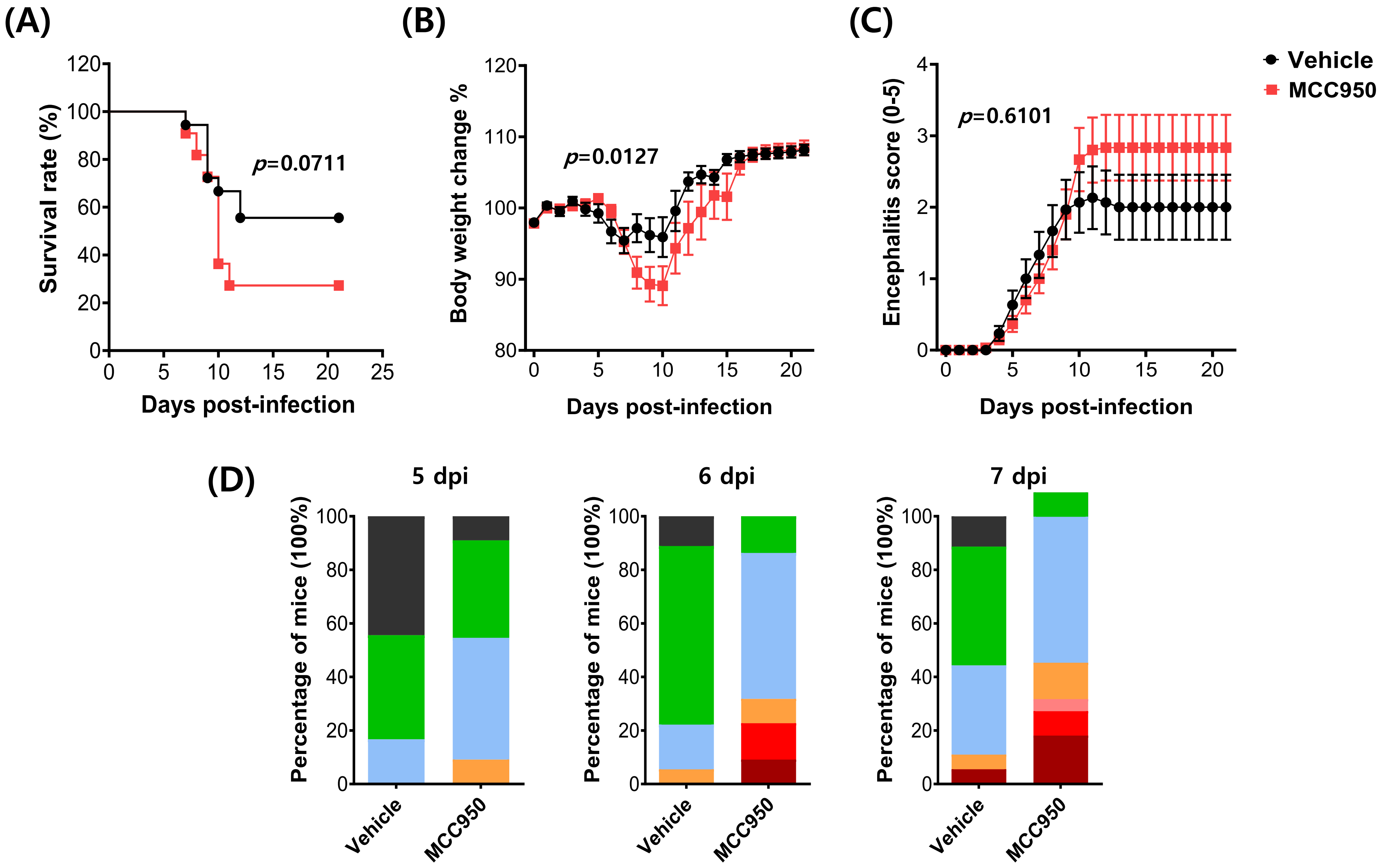

Figure S5. Enhanced susceptibility to JE by NLRP3 inhibitor MCC950. WT mice were administered with NLRP3 inhibitor MCC950 (20 mpk), commencing 1 day prior to JEV infection and continuing for a duration of 10 days. Infected mice were monitored daily for survival (A), body weight (B), and encephalitis score (C). (D) Clinical signs of MCC950-treated mice. Clinical signs of MCC950-treated mice were monitored and categorized on day 5, 6, and 7 post-infections. Statistical significance of body weight changes and encephalitis scores was determined using two-way repeated measures ANOVA, followed by Bonferroni *post hoc* tests.
