# Supplementary material for: Myeloid miR-155 deficiency exacerbates viral encephalitis by hindering M1 macrophage polarization due to impaired NLRP3 inflammasome activation in extraneural tissues
Source: Front Immunol. 2026 Jun 11;17:1818106. doi: 10.3389/fimmu.2026.1818106 (PMC13294391; doi:10.3389/fimmu.2026.1818106)
Supplement: Supplementary file 6 [file DataSheet6.pdf]

Table S1. Antibodies and reagents used for this study

Antibody	Clone	Fluorochrome	Source
CD4	RM4-5	FITC	Biolegend
CD8	53-6.7	FITC	Biolegend
CD25	7D4	FITC	Biolegend
CD44	1M7	FITC	Biolegend
CD45	30-F11	FITC	Biolegend
CD69	H1.2F3	FITC	Biolegend
CD80	16-10A1	FITC	eBioscience
CD86	GL1	FITC	eBioscience
MHC-I	28-14-8	FITC	eBioscience
MHC-II	M5/114.15.2	FITC	eBioscience
IRF5	903430	FITC	Biolegend
CD25	PC61.5	PE	Biolegend
CD44	IM7	PE	Biolegend
CD154	MR1	PE	Biolegend
CCR5	7A4	PE	eBioscience
IFN-γ	XMG1.2	PE	Biolegend
IL-2	JES6-5H4	PE	Biolegend
Ly-6C	HK1.4	PE	eBioscience
Tmem119	106-6	PE	Abcam
F4/80	BM8	PE	Biolegend
Foxp3	FJK-16S	PE	Biolegend
TLR2	6C2	PE	eBioScience
CD11b	M1/70	PerCP	Biolegend
IFN-γ	XMG1.2	PerCP	Biolegend
IL-4	11B11	PerCP	Biolegend
CD4	RM4-5	APC	Biolegend
CXCR3	CXCR3-173	APC	eBioscience
CD206	MR6F3	APC	eBioscience
IL-12/IL-23p40	C17.8	APC	Biolegend
IL-17A	eBio17B7	APC	Biolegend
TNF-α	MP6-XT22	APC	Biolegend

iNOS	CXNFT	APC	Biolegend
Ly-6G	1A8	APC	BD bioscience
F4/80	BM8	APC	eBioscience
Mouse IgG	1030-05		Invitrogen
JEV NS1 protein	ab41651		Abcam
JEV E protein	TBE-290		Abcam
NLRP3	D4D8T		Cell signaling
Caspase-1	14F468		Santa Cruz Biotech
β-actin	2D4H5		Proteintech
IL-1α	BMS6027		bioXcell
IL-1β	42410-2		bioXcell
**Reagents**			**Source**
LPS			Sigma- Aldrich
IFN-γ			eBioscience
Monensin			Sigma- Aldrich
MCC950			APExBIO
