# Supplementary material for: Myeloid miR-155 deficiency exacerbates viral encephalitis by hindering M1 macrophage polarization due to impaired NLRP3 inflammasome activation in extraneural tissues
Source: Front Immunol. 2026 Jun 11;17:1818106. doi: 10.3389/fimmu.2026.1818106 (PMC13294391; doi:10.3389/fimmu.2026.1818106)
Supplement: Supplementary file 7 [file DataSheet7.pdf]

Table S2. Specific primers for the expression of different cytokines, mucins, transcription factors, and inflammasomes in real-time qRT-PCR

Gene name	Primer sequence (5’-3’)	Gene bank ID
IFN-α	F: TCT GAT GCA GCA GGT GGG<br>R: AAG GCT CTC CAG ACT TCT GCT	NM_008333.2
IFN- β	F: TCC AAG AAA GGA CGA ACA TTC G<br>R: TGA GGA CAT CTC CCA CGT CAA	NM_010510.2
IL-1β	F: AAG TGA TAT TCT CCA TGA GCT TTG T<br>R: TTC TTC TTT GGG TAT TGC TTG G	NM_008361.4
IL-18	F: ACC GAA TTC ACT GTA CAA CCG CAG TAA TAC GGA<br>R: GCC TCT AGA GTG AAC ATT ACA GAT TTA TCC CCA	NM_001357222.1
IL-12p40	F: GGA AGC ACG GCA GCA GAA TA<br>R: AAC TTG AGG GAG AAG TAG GAA TGG	NM_001303244.1
iNOS	F: AAC GGA GAA CGT TGG ATT T<br>R: CAG CAC AAG GGG TTT TCT TC	NM_010927.4
IL-6	F: AAC GAT GAT GCA CTT GCA GA<br>R: GAG CAT TGG AAA TTG GGG TA	NM_0.31168.2
TNF-α	F: CGT CGT AGC AAA CCA CCA AG<br>R: TTG AAG AGA ACC TGG GAG TA	NM_013693.3
CXCL9	F: TGC ACG ATG CTC CTG CA<br>R: AGG TCT TTG AGG GAT TTG TAG TGG	NM_008599.4
NLRP3	F: AGC CTT CCA GGA TCC TCT TC<br>R: CTT GGG CAG CAG TTT CTT TC	NM_145827.4
NLRC4	F: CTG GAA AAG GAT GGG AAT GA<br>R: CCA AGG CAG CAT CAA TGT AG	XM_006524347.3
AIM2	F: AGG CAG TGG GAA CAA GAC AG<br>R: AAA CTT CCT GAC GCC ACC C	XM_036166274.1
ACS	F: GAA GCT GCT GAC AGT GCA AC<br>R: GCC ACA GCT CCA GAC TCT TC	XM_021168587.1
Arg1	F: GGC AAC CTG TGT CCT TTC TC	NM_007482.3

	R: ACA CGA TGT CTT TGG CAG AT	
IL-4Ra	F: ATC TGC GTG CTT GCT GGT TCT<br>R: CTG GTA TCT GTC TGA TTG GAC CG	NM_001008700.4
CD206	F: TCT TTG CCT TTC CCA GTC TCC<br>R: TGA CAC CCA GCG GAA TTT C	NM_008625.2
Fizz1	F: TCC CAG TGA ATA CTG ATG AGA<br>R: CCA CTC TGG ATC TCC CAA GA	NM_020509.4
Ym1	F: GCA GAA GCT CTC CAG AAG CAA TCC TG<br>R: ATT GGC CTG TCC TTA GCC CAA CTG	NM_009892.3
NS1	F: GGC TTA GCG CTC ACA TCC A<br>R: GCT GGC CAC CCT CTC TTC TT	AB_920399.1
β-actin	F: TGG AAT CCT GTG GGA TCC ATG AAA C<br>R: TAA AAC GCA GCT CAG TAA CAG TCC G	NM_007393.5
